# Use tumor suppressor genes as biomarkers for diagnosis of non-small cell lung cancer

**DOI:** 10.1038/s41598-020-80735-x

**Published:** 2021-02-12

**Authors:** Chuantao Zhang, Man Jiang, Na Zhou, Helei Hou, Tianjun Li, Hongsheng Yu, Yuan-De Tan, Xiaochun Zhang

**Affiliations:** 1 Precision Medicine Center of Oncology, the Affiliated Hospital of Qingdao University, Qingdao, 266003 China; 2 Dan L Duncan Comprehensive Cancer Center, Baylor College of Medicine, Houston, TX 77030 USA

**Keywords:** Cancer, Computational biology and bioinformatics, Biomarkers, Medical research, Molecular medicine, Oncology

## Abstract

Lung cancer is the leading cause of death worldwide. Especially, non-small cell lung cancer (NSCLC) has higher mortality rate than the other cancers. The high mortality rate is partially due to lack of efficient biomarkers for detection, diagnosis and prognosis. To find high efficient biomarkers for clinical diagnosis of NSCLC patients, we used gene differential expression and gene ontology (GO) to define a set of 26 tumor suppressor (TS) genes. The 26 TS genes were down-expressed in tumor samples in cohorts GSE18842, GSE40419, and GSE21933 and at stages 2 and 3 in GSE19804, and 15 TS genes were significantly down-expressed in tumor samples of stage 1. We used *S*-scores and *N*-scores defined in correlation networks to evaluate positive and negative influences of these 26 TS genes on expression of other functional genes in the four independent cohorts and found that SASH1, STARD13, CBFA2T3 and RECK were strong TS genes that have strong accordant/discordant effects and network effects globally impacting the other genes in expression and hence can be used as specific biomarkers for diagnosis of NSCLC cancer. Weak TS genes EXT1, PTCH1, KLK10 and APC that are associated with a few genes in function or work in a special pathway were not detected to be differentially expressed and had very small *S*-scores and *N*-scores in all collected datasets and can be used as sensitive biomarkers for diagnosis of early cancer. Our findings are well consistent with functions of these TS genes. GSEA analysis found that these 26 TS genes as a gene set had high enrichment scores at stages 1, 2, 3 and all stages.

## Introduction

Lung cancer is the leading cause of death worldwide^1^ which accounts for approximately 20% of deaths caused by cancer in Europe^2^ and has a high risk of recurrence^3^. In 2010, about 222,520 new cases of lung cancer were diagnosed but only 15% of patients were estimated to be alive after 5 years^4^. Based on histopathological analysis, lung cancer is divided into four major histological subtypes: small cell lung cancer, squamous cell carcinoma, adenocarcinoma and large cell carcinoma. The latter three are collectively referred to as non-small cell lung cancer (NSCLC) and account for 80% of lung cancer^5,6^. About 25 ~ 30% of patients with NSCLC had stage 1 disease and received surgical intervention alone. Despite undergoing curative surgery, more than 25% of NSCLC patients at stage 1 will die from recurrent disease within 5 years^7^. Risk factors for lung cancer identified include smoking^3^, radiation, chemical exposure, and other exposure factors^3^. Lung diseases such as chronic bronchitis, emphysema, pneumonia and tuberculosis^8^, familial tumor history^8^, and diet^9,10^ may also be considered as risk factors causing lung cancer^11^. In Western countries, 70–90% of lung cancers are attributed to cigarette smoking, whereas in Taiwan, only 7% of female lung cancer cases are associated with smoking^12,13^. Over the past 30 years, lung cancer mortality rate has increased by 464.84% in the Chinese mainland, which is much higher than the worldwide average^14^. The high mortality rate of NSCLC is partially due to lack of early detection, unclear molecular mechanism, and therapeutic methods. Also reliable clinical and molecular diagnostic and prognostic factors as well as guidelines for treatment of recurrent NSCLC stage1 have not yet been well elucidated. Identification of gene signatures and molecular pathways that are critical for development of metastasis could lead to improved therapy^7^. Advances in human genomics and proteomics have generated lists of candidate biomarkers with potential clinical values. For example, genes such as TP53^15,16^, EGFR^17,18^, KRAS^19^, PIK3CA^20^, and EML4-ALK^21^ have been identified as biomarkers for diagnosing and predicting survival outcome of lung cancers. However, most single genes are pretty unstable in expression, and hence single genes used as biomarkers do not reliably predict early lung cancers or accurately diagnose lung cancer stages^22^. Recently, many studies tried to screen gene signatures and pathways to predict survival outcome of NSCLC^7,23^ but the results are often inconsistent across studies. This is because these signature genes may provide opposite information for detection or diagnosis and prognosis due to the fact that they have up- and down-expression at different tumor stages and in different cohorts. However, a set of tumor suppressor (TS) genes chosen can be used to address these issues in prediction, diagnosis and prognosis of cancer patients because in normal cells TS genes are normally expressed to produce special proteins that result in providing ‘stop’ signals that suppress cell division, slow down the cell cycle, and mark cells for apoptosis; when TS genes are down-expressed or repressed, no or not enough the proteins provide the essential ‘stop’ signals to the cell division process and cells become cancer status^24^. In other words, in cancers, some of TS genes are down-expressed and become genes causing cancers. Ones can use this property of TS genes to find biomarkers for diagnosing early cancer. We here used differential expression analysis, gene ontology, and bioinformatics approaches to define and identify a set of 26 TS genes and used enrichment scores and network scores to evaluate these defined TS genes used as biomarkers for diagnosis of NSCLC cancer.

## Results

### Microarray data quality

To obtain correct results from differential analysis of microarray data across tumor stages, we first performed quality check (QC) of the microarray data using correlation between tumor samples and scatter plots of sample data. We randomly chose two pairs of replicate samples at each tumor stage to do QC analysis. The results show that all dots of the replicate sample pairs from stages 1, 2, and 3 were distributed around the positive diagonal line, Pearson correlation coefficients were over 0.9 (Supplementary Fig. S1), suggesting that the microarray data chosen are of high quality.

### Differential gene-expression profiles

**Figure 1:**
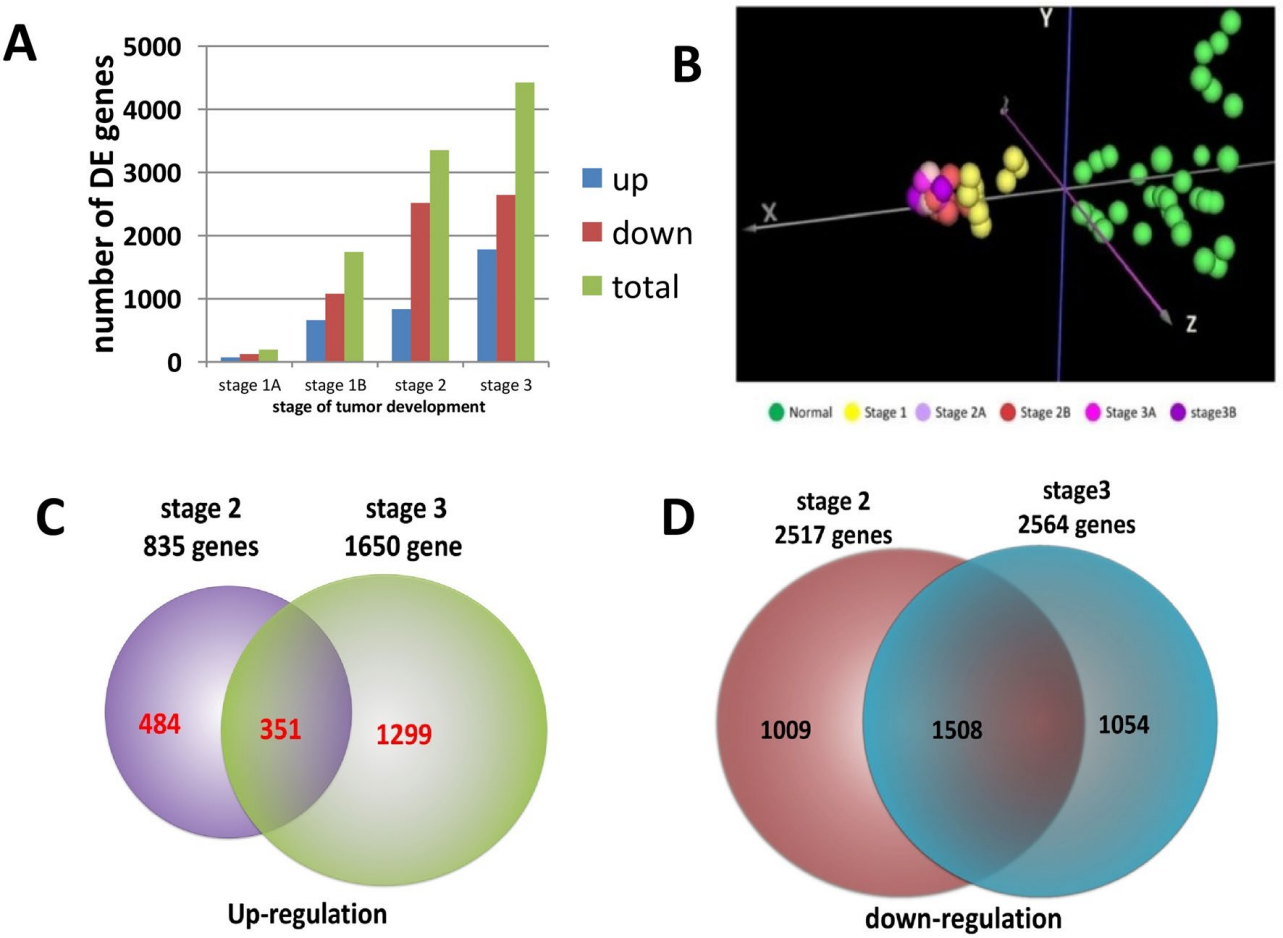
The results from gene differential expression analysis and classification of tumor stages of NSCLC patients using principle component analysis (PCA). (**A**) Number of genes differentially expressed between cancer and normal samples. Cancer and normal samples were taken from patients at stages 1–3 in Taiwan female lung cancer cohort. (**B**) PCA was used to classify tumor stages of cancer patients based on differential expression of 26 TS genes. Coordinate x is the first component, coordinates y and z are respectively the second and third components. (**C**) Numbers of up-regulated genes detected at stages 2 and 3 and of the common up-regulated genes between both stages. (**D**) Numbers of down-regulated genes detected at stages 2 and 3 and of the common down-regulated genes between both stages.

**Figure 2:**
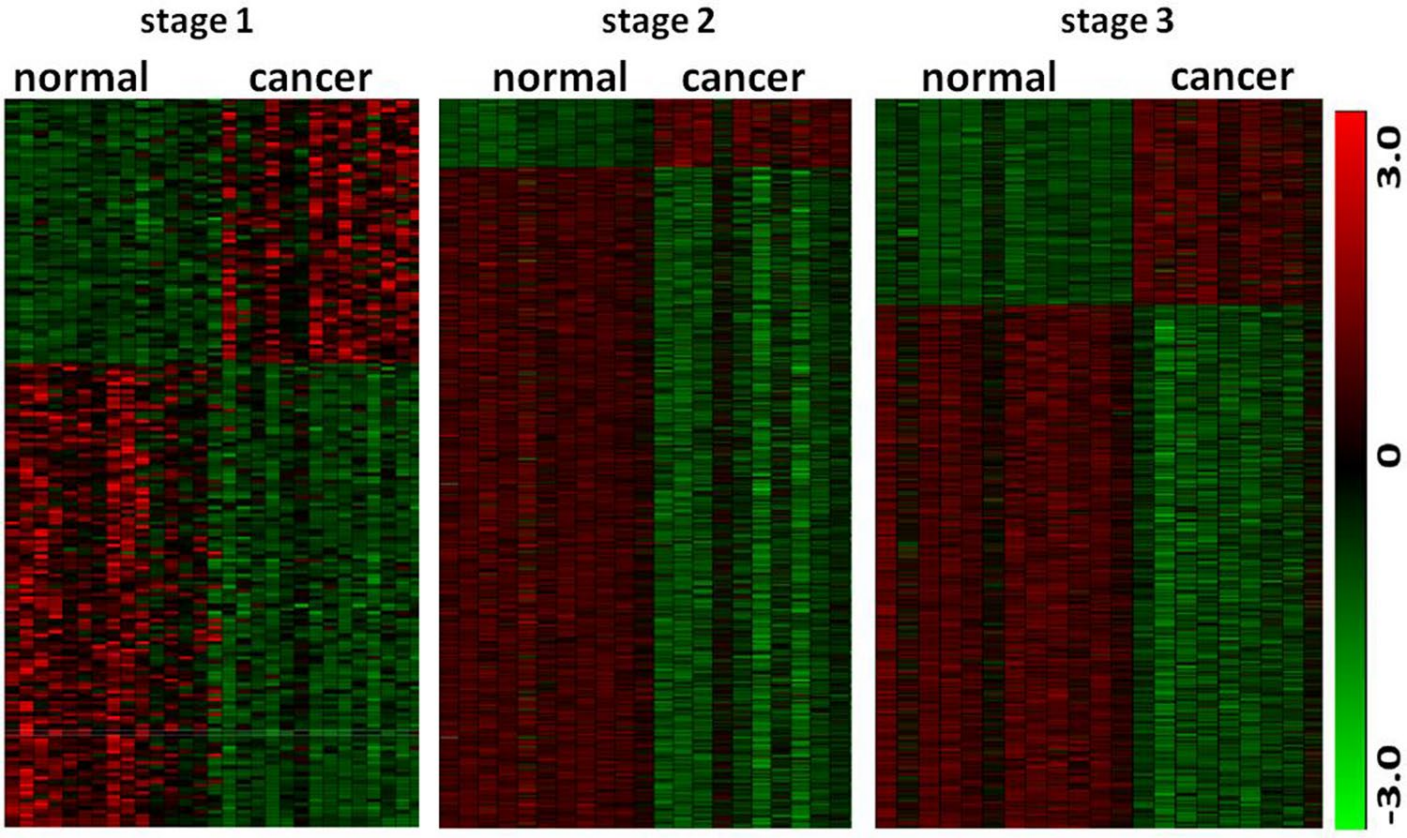
Heatmaps of gene differential expression identified at different tumor stages. Green color denotes lower expression and red color presents higher expression and black color shows no difference between normal and cancer samples. Heatmap values of genes are z-scores. At stage I, 195 probes show differential expression (DE) between normal and cancer samples. 3353 probes were detected to have differential expression at stage 2, of which 2517 probes were lowly expressed in cancer and 4424 DE probes were found at stage 3, of which 2564 probes were lowly expressed in cancer samples.

We then performed a ranking analysis of microarray (RAM)^25^ on microarray data of stages 1, 2, and 3. Figure 1A summarizes numbers of DE genes identified at stages 1, 2, and 3. Among 54,675 gene probes, only 195 (0.35%) were found to be differentially expressed at stage 1. Heatmap at stage 1 in Fig. 2 shows the differential expression of these 195 gene probes between cancer and normal samples. The result indicates that not many genes had expression change at tumor stage 1. At stage 2, however, 3352 gene probes (6%) were identified to have differential expression at FDR < 0.01, of which 2517 (75%) were down-regulated and the others (25%) were up-regulated. Compared to stage-1 heatmap, stage-2 heatmap (Fig. 2) clearly shows differential expression of these 3352 gene probes between normal lung and cancer cells. From stage 1 to stage 2, total DE gene probes increased 1700%, down-regulated gene probes increased 2029% and up-regulated gene probes increased 1176%. At stage 3, 4424 DE gene probes (8.1%) were identified, of which 2644 (60%) were down-regulated and 1780 (40%) were up-regulated. The differential expression of the 4424 gene probes between normal and cancer samples at stage 3 is shown in stage-3 heatmap (Fig. 2). From stage 2 to stage 3, total DE gene probes increased 131%, down-regulated genes increased 105% and up-regulated genes increased 213%. Among the up-regulated gene probes, we found 484 gene probes only at stage 2, 1299 only at stage 3, and 351 at both stages (Fig. 1C). For down-regulated gene probes, 1009 gene probes were detected only at stage 2, 1054 only at stage 3, and 1508 at both stages (Fig. 1D). These results indicate that abnormal expression of genes for tumor progression may start with stage 2.

### Definition of tumor suppressor genes

A list of 63 tumor suppressor genes found online^26^ was called TS gene list1. To define tumor suppressor genes in this study, we used Database for Annotation, Visualization and Integrated Discovery (David) functional annotation tool to assign these differentially expressed genes at stages 2 and 3 to different functions in human species. Then 22 and 23 tumor suppressor genes were found in functions of down-regulated DE genes at stages 2 and 3, respectively, and called TS gene list2 and list3. A gene was defined as tumor suppressor gene if this gene was found in DE genes at any stage and in TS gene list1, list2, or list3. Finally, we biologically check these TS genes using GeneCards or Wikipedia. If not, then this TS gene was excluded out of the TS gene list. Thus, 26 TS genes were found (Supplementary Table S1).

### Classification of tumor samples

We also performed principal component analysis (PCA) of the three tumor stages using data of these 26 TS genes. Figure 1B shows that normal samples (green) are clustered to the right side of Z axis (the third component) along X axis (the first component) and tumor samples at stages 1–3 are grouped into the left side of Z axis along X. Figure 1B also demonstrates that tumor samples at stage 1 (yellow) are separated from the tumor samples at stages 2–3 in the direction of normal samples by these 26 TS genes but tumor samples at substages 2A, 2B, 3A and 3B cannot be clearly separated.

### Expression of tumor suppressor genes in NSCLC

**Figure 3:**
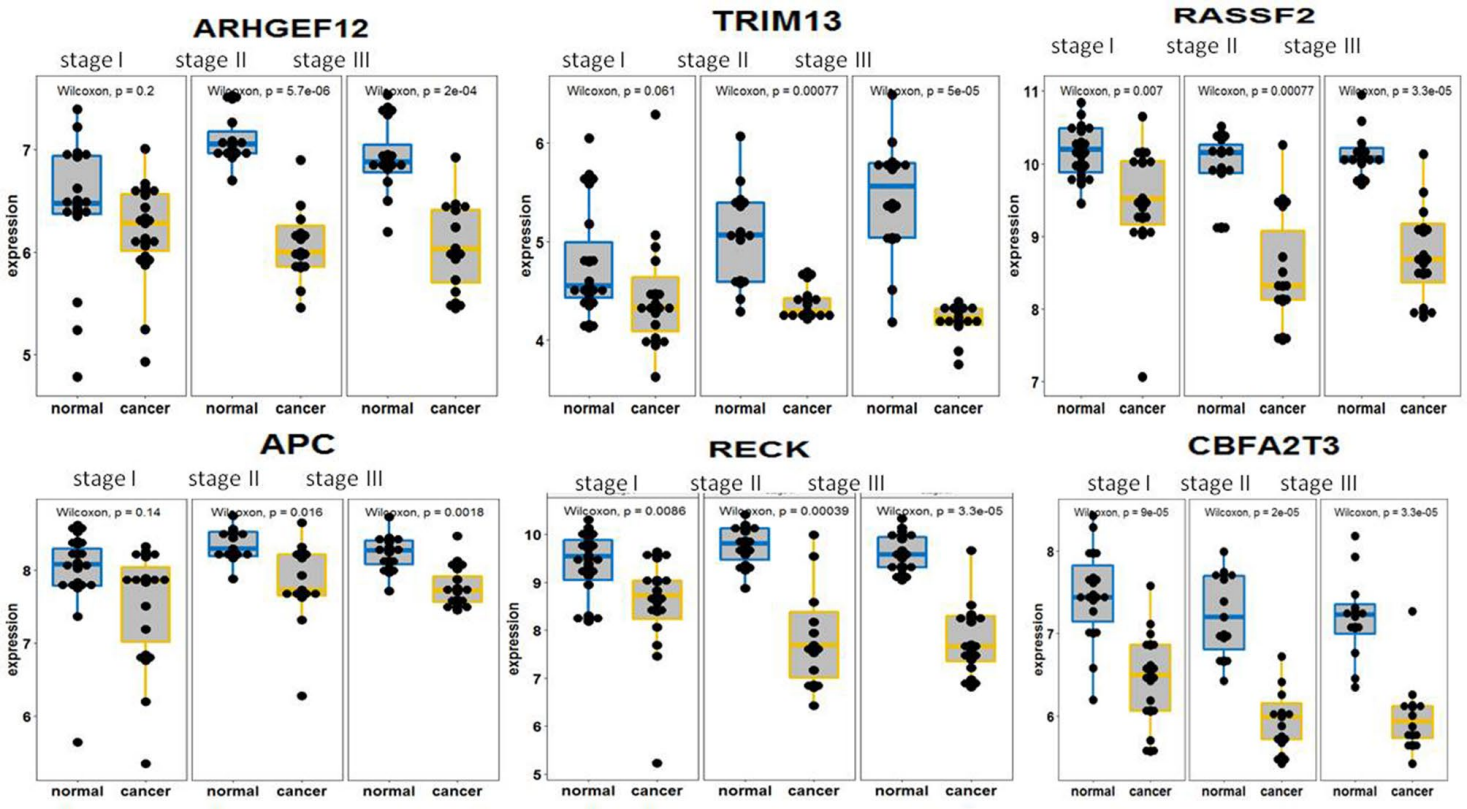
Boxplots for differential expression of tumor suppressor genes. Differential expression of 26 tumor suppressor (TS) genes between the normal and cancer samples at stages 1, 2 and 3 was displayed by boxplots where p-value is given for t-test for difference in expression of each TS gene between the normal and cancer samples. Here boxplots just show differential expression of TS genes EXT1, LIMD1, DAB2IP, DDX4, SASH1 and MCC between the normal and cancer samples at three tumor stages. The differential expression of the other 20 tumor suppressor genes was shown in supplementary Fig. S2.

We used our method (see “Methods”) to define 26 tumor suppressor (TS) genes. Since TS genes are directly associated with tumor development, it is interested in exploring dynamical changes of these TS genes in differential expression along with development of tumors. Figure 3 shows the differential expression of TS genes EXT1, LIMD1, DAB2IP, DDX5, SASH1 and MCC from tumor stage 1 to stage 3. These 6 TS genes showed much lower expression in the tumor samples than in the normal samples at stages 2 and 3. Except for that EXT1 and DDX5 were not found to be differentially expressed between the normal and tumor samples at stage1, the other 4 TS genes were down-expressed in tumor with p < 0.03 (Fig. 3). TS genes ARHGEF12, TRIM13, RASSF2, APC, RECK, and CBFA2T3 had much low expression in the tumor samples compared to the normal samples at stages 2 and 3. ARHGEF12, TRIM13 and APC had no differential expression between the normal and tumor samples at stage1 while RASSF2, RECK, and CBFA2T3 shows much lower expression in the tumor samples at stage1 than in the normal samples with p < 0.007 (Supplementary Fig. S2a). Supplementary Figures S2b and S2c show that TS genes GPC3, KLK10, KCNRG, RHOB, STARD13, CDKN1C, LATS2, RAP1A, FOXP1, TBRG1, PIK3CA and DCC were significantly lower expressed in the tumor samples than in the normal samples at stages 2 and 3. Compared to the normal samples, GPC3, KLK10, STARD13, CDKN1C, LATS2, RAP1A, FOXP1 and DCC were down-expressed in the tumor samples at stage1. In addition, NBL1 and PTCH1 were not detected to be differentially expressed between the normal and tumor samples at stages1 and 2 but were suppressed in the tumor samples at stage3 (not shown in Supplementary Fig.S2). Therefore, LIMD1, DAB2IP, SASH1, MCC, RASSF2, RECK, CBFA2T3, GPC3, KLK10, STARD13, CDKN1C, LATS2, RAP1A, FOXP1 and DCC can be used for diagnosis of early NSCLC patients. We chose two different data to validate these TS genes selected. Because GSE18842 had the same microarray platform with GSE19084, we used probeid to retrieve expression data of 26 TS genes from GSE18842 and made a heatmap in R environment (https://www.r-project.org). The result was shown in Supplementary Figure S3. The heatmap shows that these selected TS genes were really also down-expressed in the 45 cancer samples (Supplementary Fig. S3). Interestingly, RNA-seq RPKM data retrieved from cohort GSE40419 also demonstrates that these TS genes were down-expressed in either smoking or non-smoking NSCLC samples (Supplementary Fig. S3).

### Evaluation of TS genes as biomarkers for diagnosis of NSCLC cancer

#### *S*-score analysis

**Figure 4:**
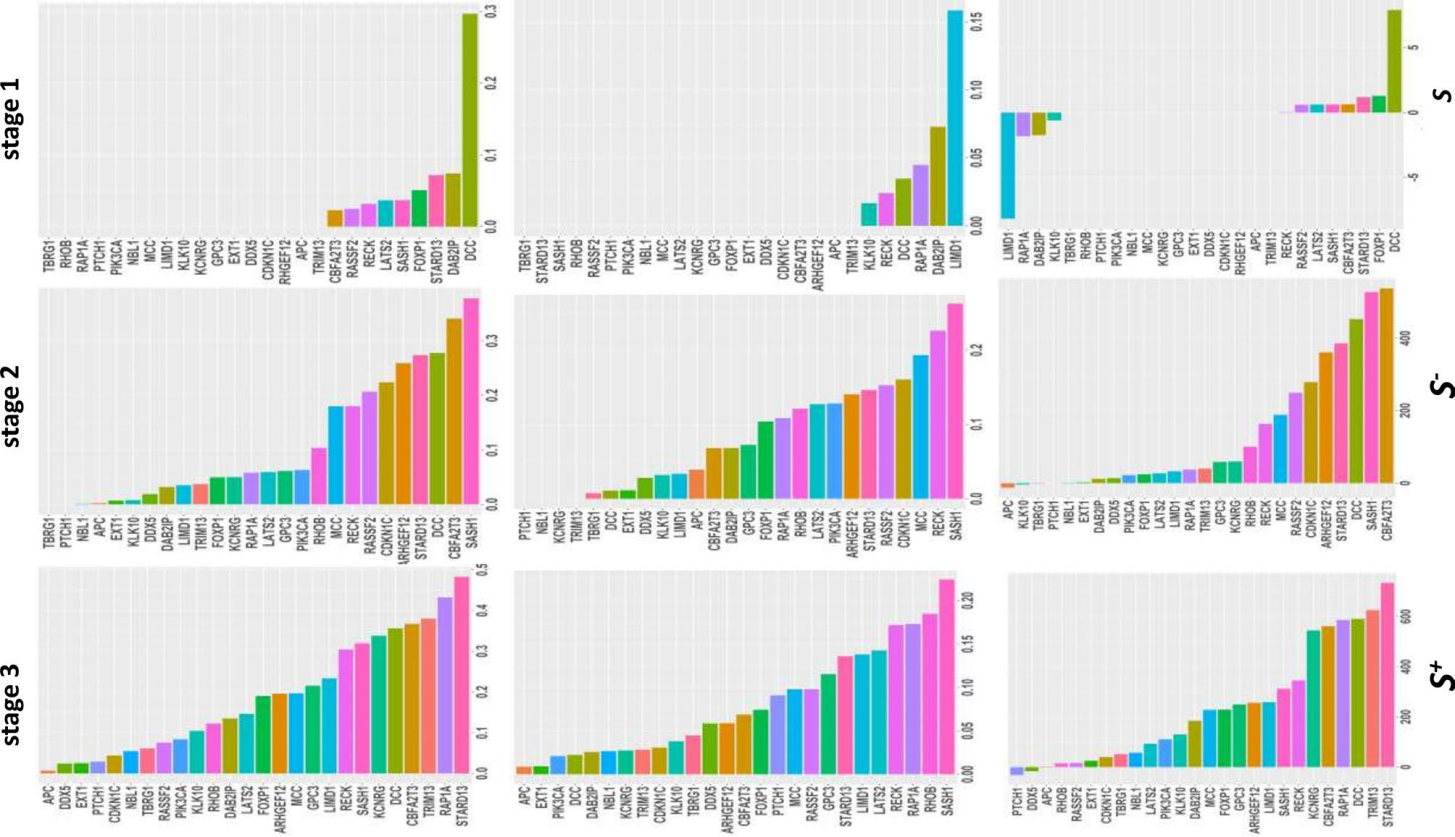
Histogram plots for ${S}^{+}$, ${S}^{-}$ and S of TS genes. Correlation coefficients were used to measure roles of TS genes in regulation of expression of the other genes. TS genes with negative correlation coefficients $\le$ -T (T is a threshold value with Bonferroni adjusted p < 0.05, see “Methods” for calculation of T value) have a negative role that up-regulates expression of genes while those with positive correlation coefficients $\ge$ T have a positive role that down-regulates expression of the other genes. *S*-score is defined as sum of all correlation coefficients larger than or equal to a given positive threshold or less than or equal to a given negative threshold for a TS gene *i*: ${S}_{i}$ = ${\sum }_{j=1}^{m}{r}_{ij}||r|\ge T$ where *j*=1, 2, ..., *m* and *i* = 1, 2, ..., *n.*  Here *n* and *m* are numbers of DE and TS genes, respectively. ${S}^{+}$ is proportion of sum of all correlation coefficients larger than or equal to a given threshold to sums of correlation coefficients larger than zero for a TS gene *i*: ${S}_{i}^{+}=({\sum }_{j=1}^{m}{r}_{ij}|r\ge T)/({\sum }_{j=1}^{m}{r}_{ij}|r>0)$. Similarly, ${S}^{-}$ is defined as ${S}_{i}^{-}=({\sum }_{j=1}^{m}{r}_{ij}|r<-T)/({\sum }_{j=1}^{m}{r}_{ij}|r<0)$ where T = 0.62 for stage 1, 0.765 for stage 2, and 0.75 for stage 3.

We here used correlation of TS genes found at a tumor stage with the DE genes identified at the same stage to define S-scores (see “Methods”) and used *S*-scores to explore the role of a TS gene in regulating expression of functional genes. With correlation coefficients $>$ T (T is a threshold for selection of correlation coefficients with Bonferroni adjusted p-values < 0.05, here T = 0.62 for stage 1, 0.765 for stage 2, and 0.75 for stage 3), we calculated ${S}^{+}$ of 26 TS genes at stages 1, 2 and 3. The results were plotted in Fig. 4. At stage 1, Fig. 4 shows that DCC had the largest ${S}^{+}$, indicating that DCC was the strongest TS gene to positively impact on gene down-expression at stage 1. Besides DCC, CBFA2T3, FOXP1, SASH1, LATS2, and DAB2IP had ${S}^{+}$ > 0, suggesting that the six defined TS genes had roles in regulating down-expression of the other functional genes in early lung cancer. As seen in the boxplots, these 6 TS genes were significantly down-expressed in lung cancer (Fig. 3 and Supplementary Fig. S2); hence, their expression may be used to diagnose early NSCLC patients. At stage2, SASH1 had the strongest positive network effect (network effect is such an effect that a TS gene impacts on a set of the other functional genes in expression by correlation network) on gene down-expression in cancer and STARD13, RECK, CBFA2T3, ARHGEF12, CDKN1C, and RASSF2 also had larger ${S}^{+}$, implicating that these 6 TS genes were stronger TS genes to positively impact on down-expression of a lot of the other functional genes in cancer. In addition, MCC, RHOB, RAP1A, LATS2, GPC3 had ${S}^{+}$ $\ge$ 0.2, and hence played a medium positive role in gene down-expression in cancer. PIK3CA, LIMD1, KCNRG, DDX5, DAB2IP, APC, TRIM13, KLK10, NBL, EXT1, and TBRG1 had ${S}^{+}$ < 0.1, so they had weak network effects on gene expression in stage-2 cancer (Fig. 4). PTCH1 was not positively correlated with any DE genes in cancer. At stage 3, RAP1A and STARD13 showed the largest ${S}^{+}$. Next, SASH1 and RECK had ${S}^{+}$ over 0.5. At stage 3, RASSF2 and ARHGEF12 had ${S}^{+}$ less than 0.3, while KCNRG, DCC, TRIM13 and LIMD1 increased ${S}^{+}$ from less than 0.2 at stage 2 to more than 0.3 (Fig. 4). APC, EXT1 and DDX5 still had small ${S}^{+}$. These suggest that RAP1A, STARD13, SASH1, RECK, and CBFA2T3 had big positive network effects on down-expression of genes in stage-3 cancer. LATS2, MCC, DCC, RHOB, TRIM13, LIMD1, and GPC3 also strongly and positively regulated gene down-expression in cancer. APC, PTCH1, EXT1, TBRG1, NBL1, PIK3CA, and KLK10 still had very weak network effects.

${S}^{-}$ was given by setting correlation coefficients < -T (see “Methods”) and hence used to define a negative network effect of a TS gene on regulating gene up-expression. At stage 1, only DCC, DAB2IP, LIMID1 and RAP1A had weak negative network effects on up-expression of genes. These four TS genes were very significantly down-expressed in cancer at stage 1 with p-value $\le$ 0.009 (Fig. 3 and Supplementary Fig. S2c). Figure 4 shows that both stages 2 and 3 had very similar TS gene ${S}^{-}$ profiles. First, TBRG1, PICH1, NBL1, TRIM13 and APC did not impact gene up-expression in cancer. Second, SASH1 was the strongest TS gene acting on gene up-expression in cancer. Next, RECK, STARD13 and RASSF2 were stronger TS genes to negatively regulate gene up-expression. MCC, CBFA2T3, CDKN1C, ARHGEF12, PIK3CA and RHOB had approximately negative expression association with the other functional genes.

*S*-score is defined by the sum of correlation coefficients of a TS gene larger than T or smaller than -T (“Methods”). If a TS gene has *S* > 0, then the TS gene has larger positive network effect on gene down-expression than negative one on gene up-expression in cancer. If a TS gene has *S* < 0, then it has larger negative network effect on gene up-expression than positive one on gene down-expression in cancer. If a TS gene has S = 0, then it does not act on gene expression or balances between up- and down-regulations of gene expression. At stage 1, LIMD1, RAP1A and DAB2IP had negative network effects on gene up-expression in early cancer, while SASH1, CBFA2T3, LATS2, RECK, FOXP1 and DCC had positive network effects on gene down-expression, in other words, down-expression of these 6 TS genes resulted in or associated with down-expression of genes in early cancer. Interestingly, 9 TS genes with *S* > 0 or *S* < 0 were found to be indeed significantly down-expressed in cancer at stage 1, while the other TS genes such as EXT1, KLK10, NBL1, RHOB, PTCH1 that were not found to be down-expressed in early cancer had *S* of zero, that is, these of being not differentially expressed between normal and cancer samples at stage 1 were not found to be correlated with other functional genes in expression in early cancer. Indeed, except for that PTCH1, KLK10, TBRG1, NBL1 and EXT1 did not act on gene expression in stage-2 cancer, the other 21 TS gene had larger positive network effects on gene expression than negative network effects in cancer. At stage 3, however, all these 26 defined TS genes have *S* > 0, suggesting that all these 26 TS genes had larger positive network effects on gene down-expression in cancer than negative network effects on gene up-expression even though APC, EXT1 and DDX5 had very small *S*-scores.

In order to validate impacts of TS genes on gene expression in cancer, we used T = 0.474 to calculate their ${S}^{+}$, ${S}^{-}$ and *S* in cohort GSE18842^27^. The results were summarized in supplementary Figure S4. Similarly, STARD13, RECK, RAP1A, LATS2, LIMD1, RASSF2, CBFA2T3, SASH1, DDX5, ARHGEF12, CDKN1C, and RHOB had ${S}^{+}$ > 0.4 and ${S}^{-}$ > 0.3, indicating that in cohort GSE18842 these TS genes had larger positive and negative network effects on expression of genes in cancers. PIK3CA, APC, KLK10 and PTCH1 still weakly acted on gene down-expression and did not impact gene up-expression. Except for that GPC3 had negative *S*-score, all the other 25 TS genes had positive *S*-score, demonstrating that in cohort GSE18842^27^ these TS genes also had much larger positive network effects on gene expression than negative ones. In particular, CBFA2T3, ARHGEF12, SASH1 and STARD13 still had the strongest impact on gene down-expression. Interestingly, in the RNA-seq data from cohort GSE40419^28^ without smoking history, ${S}^{+}$, ${S}^{-}$ and *S* obtained from correlation coefficients selected by T = 0.538 or T = − 0.538 show similar results (Supplementary Fig.S4). Likewise, all these 26 TS genes had ${S}^{+}$ > 0. Similarly, KLK10, KCHRG, EXT1, PIK3CA, and PTCH1 also had ${S}^{-}$ = 0. Except for that RAP1A and RASSF2 had negative *S*, all the other 24 defined TS genes had positive *S*-scores, demonstrating that in no smoking cohort GSE40419^28^, TS genes had much larger positive network effects on gene expression than negative ones, or, down-expressed genes due to down-expression of these TS genes were many more than up-expressed genes resulted from their down-expression in cancers. Supplementary Figure S4 displays ${S}^{+}$, ${S}^{-}$ and *S* profiles of these 26 TS genes in smoking cohort GSE40419^28^. Compared to these score profiles of TS genes in no smoking cohort, we found that smoking strongly impacted ${S}^{+}$, ${S}^{-}$ and *S* scores. For example, GPC3, CDKN1C, LIMD1 had ${S}^{+}$ larger than 0.4 in no smoking cohort but decreased to less than 0.2 in smoking cohort. APC, DDX5, CDKN1C, LIMD1, TBRG1, FOXP1 decreased ${S}^{-}$ to 0.05 in the smoking cohort, while MCC, RAP1A, STARD13, NBL1, increased ${S}^{-}$ from 0.15 in no smoking cohort to 0.35 in smoking cohort. However, except for that LATS2, RASSF2, RAP1A, NBL1 and RHOB had negative *S*-scores, the other 21 TS genes also had positive *S*-score, demonstrating that in smoking cohort GSE40419, TS genes had much larger positive network effects on gene expression than negative ones, or, down-expressed genes due to down-expression of these TS genes were many more than up-expressed genes resulted from their down-expression in cancer.

#### *N*-score analysis

**Figure 5:**
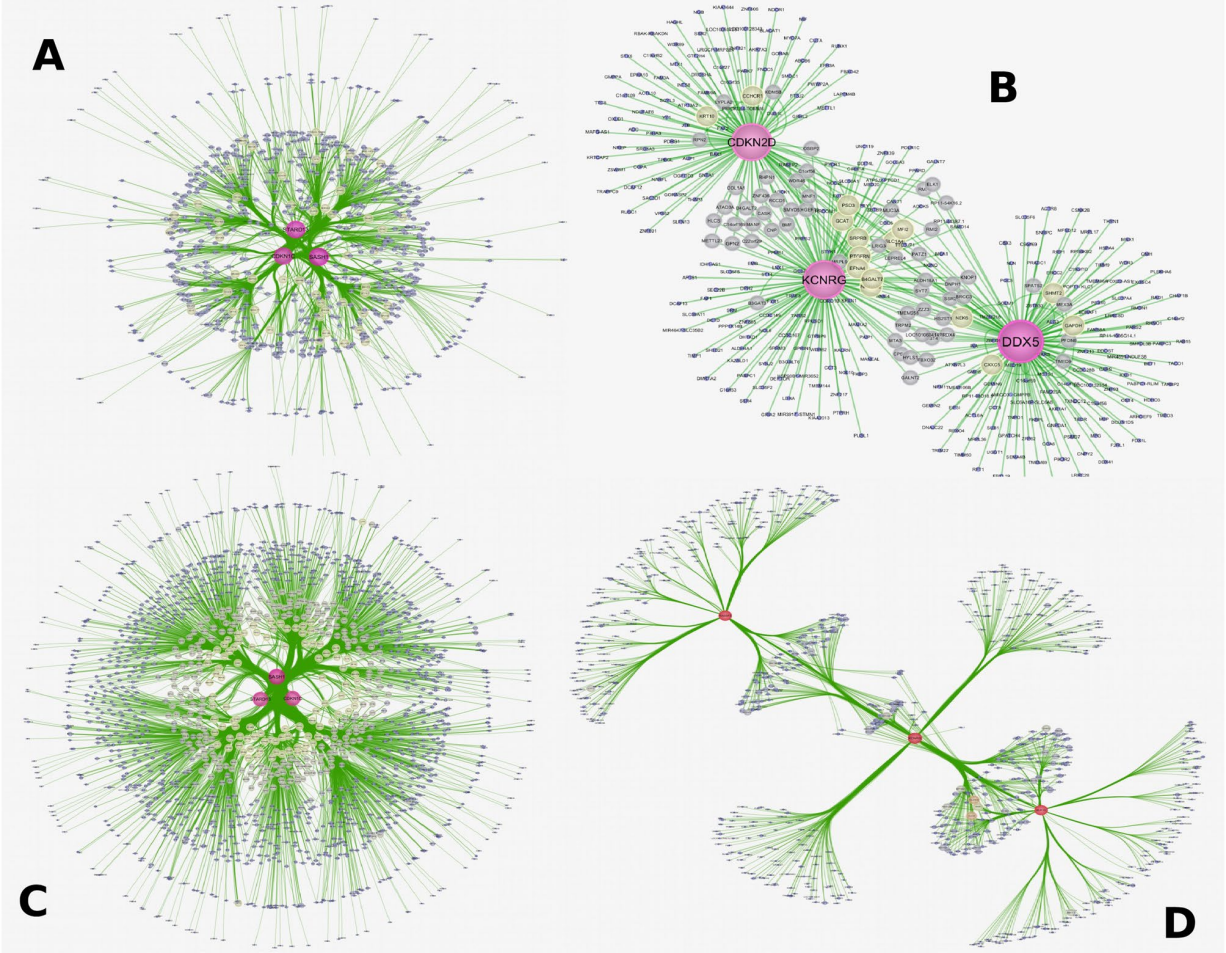
Correlation networks of tumor suppressor genes with DE genes. Examples for simple and complicated networks respectively constructed with correlations of three strong and weak TS genes with DE genes. (**A**) Negative correlation network of strong TS genes STARD13, CDKN1C, and SASH1 with DE genes at stage 2. (**B**) Negative correlation network of weak TS genes CDKN2D, KCNRG and DDX5 with DE genes at stage 2. (**C**) Positive correlation network of strong TS gene STARD13, CDKN1C, and SASH1 with DE genes at stage 2. (**D**) Positive correlation network of weak TS genes CDKN2D, KCNRG and DDX5 with DE genes at stage 2.

**Figure 6:**
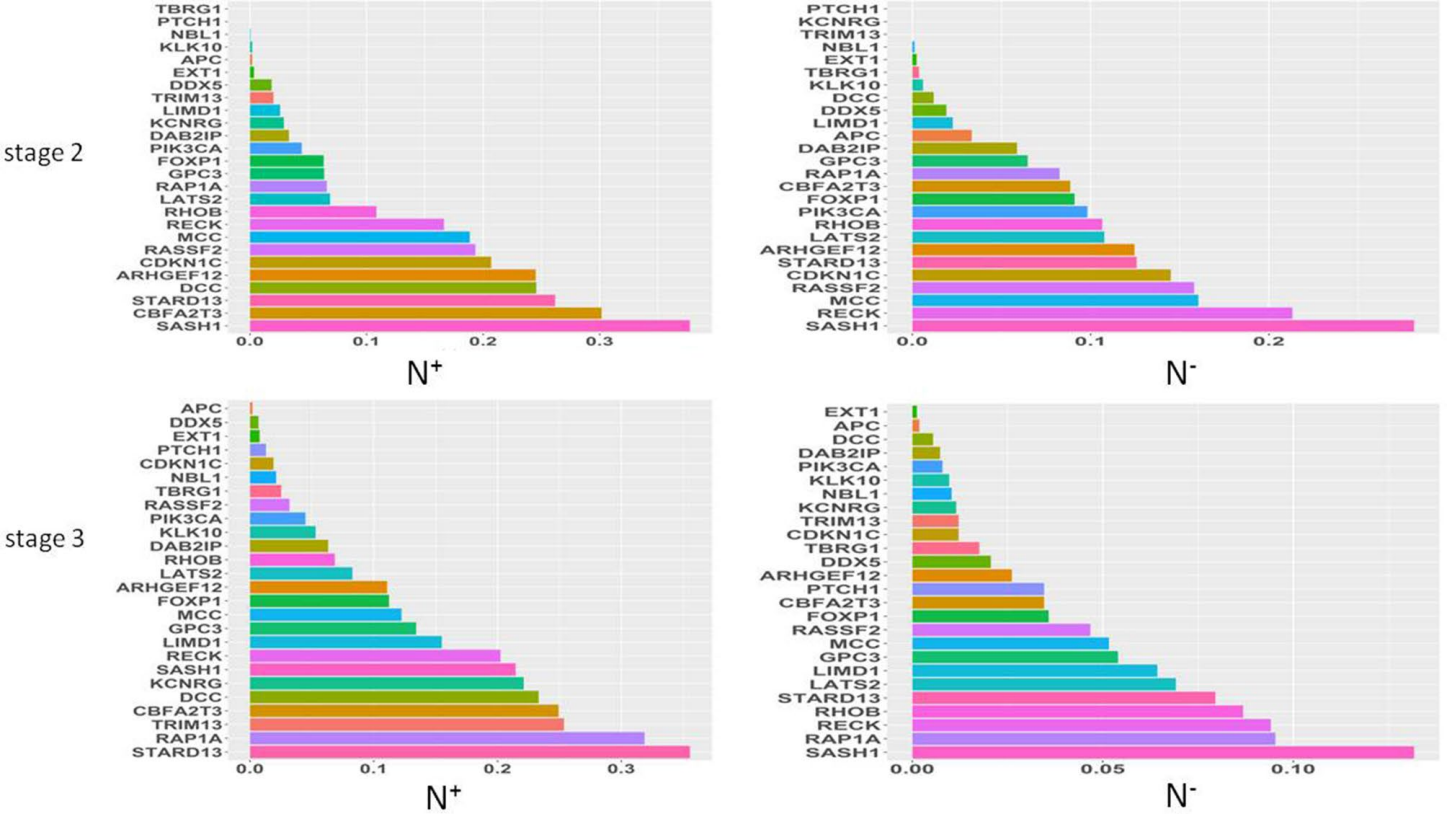
Histogram plots for ${N}^{+}$ and ${N}^{-}$ of TS genes. Network score or N-score is defined as ratio of node number shared with a TS gene and all the other TS genes in a network constructed at a given significance level to that in a network constructed at insignificance level(r > 0 or r < 0). *N*-score is given by ${N}^{+}$ and ${N}^{-}$(see “Methods” for detail definition and calculation). ${N}^{+}$ is used to measure accordant effects of a TS gene in a positive correlation networks on down-expression of genes. ${N}^{-}$ is used to measure discordant effects of a TS gene in a negative correlation networks on up-expression of genes.

As examples, we here employed correlation coefficients of three strong TS genes and three weak TS genes defined by using *S*-scores with DE genes identified at stage 2 in cohort GSE19804^1^ to respectively construct positive and negative correlation networks (Fig. 5). Strong TS genes SASH1, STARD13 and CDKN1C had very complicated positive and negative networks (Fig. 5A,C) in which these TS genes commonly shared many more DE genes (double-line or multi-line nodes) than they unshared DE genes (single-line nodes), while weak TS genes DDX5, KCNRG and CDKN2C had very simple positive and negative correlation networks (Fig. 5B,D), that is, these three weak TS genes commonly shared less DE genes (double-line or multi-line nodes) than they unshared DE genes(single-line nodes). Summarily, strong TS genes had not only stronger network effects (strongly connected more DE genes) but also stronger synergy effects (commonly shared more DE genes) than weak TS genes. To evaluate TS genes in synergy effects on commonly regulating gene expression, we here proposed *N*-scores (or network scores) to compare these TS genes (see “Methods”). Figure 6 shows the *N*-scores of 26 TS genes at tumor stages 2 and 3. ${N}^{+}$ was distributed in 0 ~ 0.4 at both stages 2 and 3. However, ${N}^{-}$ was reduced from ~ 0.38 at stage 2 to ~ 0.16 at stage 3. At stage2, SASH1 had the largest ${N}^{+}$ (0.40) and ${N}^{-}$ (0.38), indicating that SASH1 had the strongest positive and negative accordant/discordant effects on expression regulation of genes in cancer. The next are CBFA2T3 (${N}^{+}$= 0.3) and STARD13 (${N}^{+}$= 0.22) or RECK (${N}^{-}$= 0.22) and MCC (${N}^{-}$= 0.17). TBRG1 and PTHC1 had ${N}^{+}$ of zero and PTCH1, KCNRG and TRIM13 had ${N}^{-}$ of zero. These TS genes did not positively and/or negatively impact expression of genes in cancer. TS genes NBL1, KLK10, APC, and EXT1 had very small ${N}^{+}$ (< 0.01) and ${N}^{-}$ (< 0.01). Therefore, these genes were weak TS genes. At stage 3, STARD13, CBFA2T3, RECK, and SASH1 had strong accordant/discordant effects on expression of genes in cancer, while APC, EXT1, NBL, KLK10, and PTCH1 had very weak accordant/discordant impacts on expression of genes in cancer. Interestingly, DCC had larger ${N}^{+}$ (> 0.2) but very small ${N}^{-}$ (< 0.01) at both stages 2 and 3, implicating that DCC was not correlated with up-expressed genes in cohort GSE19804^1^. In cohort GSE18842, ${N}^{+}$ and ${N}^{-}$ profiles were similar (Supplementary Fig. S5a): DCC, TBRG1, and TRIMP1 had ${N}^{+}$ and ${N}^{-}$ of zero. RAP1A, MCC, PIK3CA, DDX5, and FOXP1 had very small ${N}^{+}$ (< 0.05) and ${N}^{-}$ (< 0.01). Stronger TS genes STARD13, CBFA2T3, ARHGEF12, and RHOB also had larger ${N}^{+}$ ($\ge 0.3)$ and ${N}^{-}$ ($\ge 0.3)$. Different from cohort GSE19804, the weak TS genes KLK10, NBL1, and APC became strong TS genes with ${N}^{+}$ > 0.4 and ${N}^{-}$ > 0.4 (Supplementary Fig. S5a). Inversely, stronger TS genes RAP1A, and MCC at stage 3 in cohort GSE19804 became very weak TS genes with ${N}^{+}$ < 0.05 and ${N}^{-}$ <0.01. ${N}^{+}$ profile in cohort GSE40419^28^ (Supplementary Fig. S5b) is pretty similar to that at stage 3 in GSE19804. For example, EXT1, PTCH1, KLK10 and APC were weak TS genes with small ${N}^{+}$ in these two cohorts, while strong TS genes RECK, SASH1, CBFA2T3, STARD13, DCC, and MCC in GSE19804 were still strong TS genes in GSE40419 with ${N}^{+}$ > 0.3 (Supplementary Fig. S5b). ARHGEF12, TRIM13, RAP1A, KCNRG, LATS2, RHOB and RASSF2 varied in these two cohorts. ${N}^{-}$ profile was similar to ${N}^{+}$ profile in GSE40419. RECK, SASH1, CBFA2T3, STARD13, FOXP1, CDKN1C, and LATS2 were strong TS genes with ${N}^{+}$ > 0.3 and ${N}^{-}$ > 0.1. Like in GSE19804, DCC also had larger ${N}^{+}$ (> 0.4) but small ${N}^{-}$ (< 0.05).

#### Score accumulation profiles

**Figure 7:**
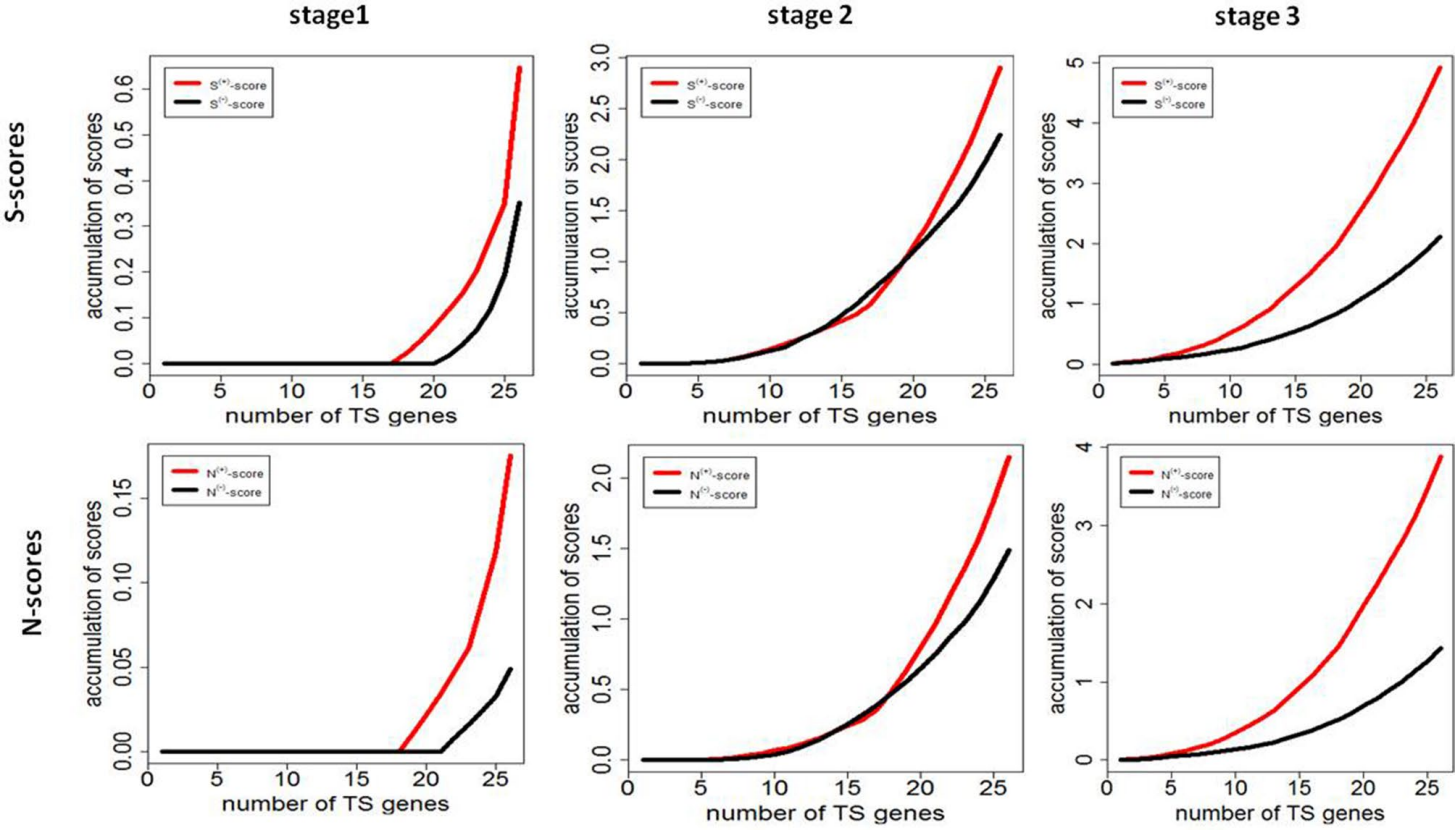
${S}^{+}$ and ${S}^{-}$ and ${N}^{+}$ and ${N}^{-}$ accumulation profiles. Accumulations of *S*-scores and *N*-scores are calculated by ${X}_{1}^{a}={N}_{1}^{a}$, ${X}_{2}^{a}={N}_{1}^{a}+{N}_{2}^{a}$, …, ${X}_{n}^{a}={N}_{1}^{a}+{N}_{2}^{a}+\cdots +{N}_{n}^{a}$. ${Y}_{1}^{a}={S}_{1}^{a}$, ${Y}_{2}^{a}={S}_{1}^{a}+{S}_{2}^{a}$, …, ${Y}_{n}^{a}={S}_{1}^{a}+{S}_{2}^{a}+\cdots +{S}_{n}^{a}$ where $a$ = “ + ” or “-”, ${N}_{1}^{a}\le {N}_{2}^{a}\le \cdots \le {N}_{n}^{a}$ and ${S}_{1}^{a}\le {S}_{2}^{a}\le \cdots \le {S}_{n}^{a}$ are ranked N-score and S-score lists from the smallest to the largest. Here, 1, 2, …, *n* in *X* or *Y* are code for *n* TS genes. For example, from Fig. 4, ${S}_{1}^{+}={S}_{PTCH1}^{+}$ and ${S}_{26}^{(+)}$ = ${S}_{SASH1}^{+}$ at stage 2. ${X}_{1}^{a}$ is *N*-score for the first TS gene, ${X}_{2}^{a}$ is sum of *N*-scores for the first and second TS genes, and so on. Plot these score accumulations along numbers of TS genes to form an accumulation curve. Stages 2 and 3 are tumor stages 2 and 3. The data for calculating *S*-scores and *N*-scores are from stage2 and 3, respectively. Difference between ${X}^{+}$ and ${X}^{-}$ or between ${Y}^{+}$ and ${Y}^{-}$ may be related to development of tumor stage.

To globally compare scores of negative and positive networks, we calculated score accumulations from the smallest value to each ordered value (${X}_{i}^{a}={\sum }_{k=1}^{i}{S}_{k}^{a} \, \mathrm{ where\ }$ ${S}_{1}^{a}\le {S}_{2}^{a}\le \dots \le {S}_{n}^{a}$ or ${Y}_{i}^{a}={\sum }_{k=1}^{i}{N}_{k}^{a}$ where ${N}_{1}^{a}\le {N}_{2}^{a}\le \dots \le {N}_{n}^{a}$, *i* is an ordered number, *i*=1, 2, ..., N and *a*=+/−) and plotted these score accumulations to form an accumulation curve along numbers of TS genes. The plots are shown in Fig. 7 where we found that at stage 1 in cohort GSE19804, ${S}^{+}$ and ${S}^{-}$ accumulations are zero when numbers of TS genes < 18 and the largest difference between ${S}^{+}$ and ${S}^{-}$ accumulations is 0.32. At stage 2, ${S}^{+}$ and ${S}^{-}$ accumulations were almost the same along numbers of TS genes < 18. Over 18 TS genes, ${S}^{+}$ accumulation became larger than ${S}^{-}$ accumulation and the largest difference between both is 0.75. At stage 3, however, ${S}^{+}$ accumulation was larger than ${S}^{-}$ accumulation at all points of which numbers of TS genes are larger than 6 and difference between them became larger and larger as number of TS genes increased and the largest difference is 3.0. This means that at stage 3, more than 6 TS genes commonly connected many more down-expressed genes than they commonly connected up-expressed genes in cancer. Supplementary Figure S7 shows that in GSE40419 non smoking cohort, ${S}^{+}$ and ${S}^{-}$ accumulation curve profile is very similar to that at stage 3 in GSE19804 and the largest difference between ${S}^{+}$ and ${S}^{-}$ accumulations is 4.8. This allows us to infer that these lung tumor samples in GSE40419 were in between tumor stages 3B and 4. In cohort GSE18842, ${S}^{+}$ and ${S}^{-}$ accumulation curve profile is in between stages 2 and 3, that is, the largest difference between ${S}^{+}$ and ${S}^{-}$ accumulation curves is 1.0, larger than 0.75 at stage 2 but much smaller than 3.0 at stage 3, inferring that most of tumor patients in cohort GSE18842 were at stage 2 and small part of patients were at stage 3. To confirm the inference, we applied our *S*-scores and *N*-scores to a microarray data (GSE21933^29^) from platform GPL6254 where there were 21 pairs of normal samples and NSCLC tumor samples. We took the data of 3 tumor stage-2 samples and 6 tumor stage-3 samples and all normal samples to do *S*-score and *N*-score analyses. The results, as we expected, shows that ${S}^{+}$ and ${S}^{-}$ accumulation curve profile is between those in GSE18842 and at stage 2 in GSE19804 (Fig. 7), and the largest difference between ${S}^{+}$ and ${S}^{-}$ accumulations is 1.5, demonstrating the above inference of tumor stages of patients in GSE18842. From Fig. 7 and Supplementary Figure S7, we found that ${N}^{+}$ and ${N}^{-}$ accumulation curve profile is pretty similar to ${S}^{+}$ and ${S}^{-}$ accumulation curve profile in all cohorts though *S*-score accumulation scale is much larger than N-score accumulation scale.

#### Gene set enrichment analysis (GSEA)

**Figure 8:**
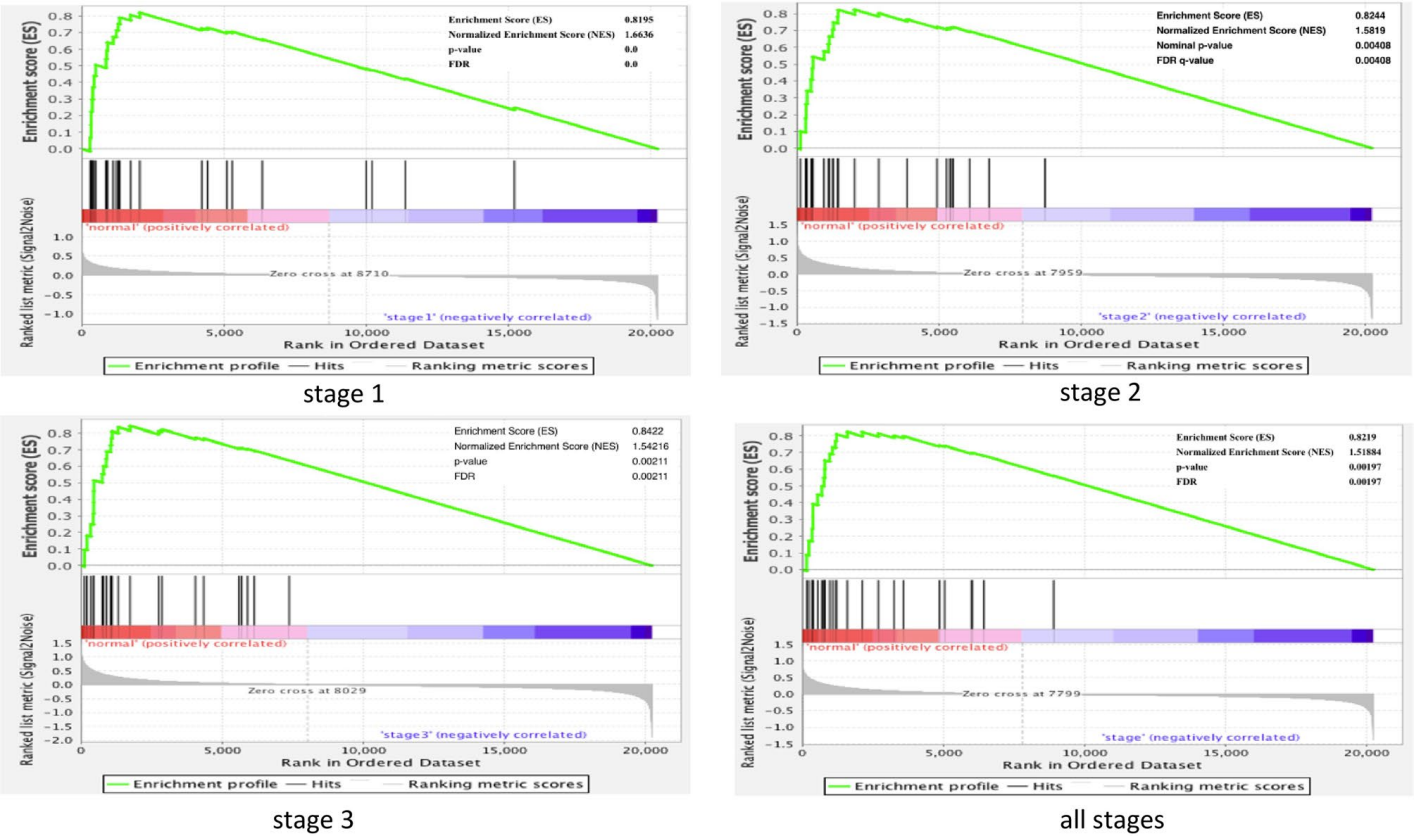
GSEA analysis of TS genes. GSEA plot shows profile of the running ES scores & positions of GeneSet members on the rank ordered List of genes. 26 TS genes were setup as a gene set, samples from Taiwan female NSCLC cohort were used as phenotype data and Human_AFFY_HG_U133_ MSigDB.v7.1.chip was used as annotation file. GSEA analysis was respectively performed on microarray datasets at stages 1, 2, 3 and all stages. Permutation was performed among samples for 1000 times for calculating p-value. FDR is used for multiple tests^38^. Stage 1: 38 normal samples and 15 cancer samples. Stage 2: 38 normal samples and 11 cancer samples. Stage 3: 38 normal samples and 7 cancer samples. All stages: 38 normal samples and 38 cancer samples.

To furthermore demonstrate impact of the defined 26 TS genes on development of NSCLC cancer, we performed GSEA^30^ to analyze enrichment of the 26 TS genes as a gene set on gene expression data GSE19804 at tumor development stages 1, 2 and 3, and all stages, respectively. We separately performed permutations among samples and among genes to calculate p-value for enrichment analysis. The results obtained from 1000 permutations among samples and among genes are shown in Fig. 8 and Supplementary Figure S8, respectively. All the GSEA results show very similar enrichment profiles at tumor development stages 1–3 and all stages. All the defined TS genes were enriched on positive correlation (down expression of TS genes) and the maximum enrichment score was 0.8 with p-value < 0.002. The results indicate that the 26 TS genes are of strong information for biomarkers to predict or diagnose NSCLC.

## Discussion

*S*-scores are a metric for a role or an impact of a TS gene on expression of the other functional genes by measuring correlation expression of this TS gene with the other functional genes. *N*-scores are another metric for a synergy effect of a TS gene with the other TS genes on expression of the other functional genes. Although *S*-scores and *N*-scores are two different types of metrics, they depend upon numbers of DE genes correlated with TS genes in expression under a given significance level. This is why ${N}^{+}$ and ${N}^{-}$ accumulation profile is very similar to ${S}^{+}$ and ${S}^{-}$ accumulation profile in all collected gene expression datasets. If a TS gene correlates with many other functional genes in expression, then it would have large ${S}^{+}$ or large ${S}^{-}$. For TS genes, ${S}^{+}$ $\ge {S}^{-}$ and ${N}^{+}$ $\ge {N}^{-}$. Therefore, ${S}^{+}$ and ${N}^{+}$ accumulations are larger than or equal to ${S}^{-}$ and ${N}^{-}$ accumulations. This phenomenon was observed in two data types (microarray and RNA-seq), two platforms (GPL570 and GPL6254), 4 cohorts (GSE19804, GSE18842, GSE21933 and GSE40419), and at two tumor stages. Interestingly, difference between ${S}^{+}$ and ${S}^{-}$ accumulations or between ${N}^{+}$ and ${N}^{-}$ accumulations along number of TS genes became larger and larger as tumor stage developed.

A TS gene is a strong TS gene if it has larger ${S}^{+}$ and ${N}^{+}$ and/or larger ${S}^{-}$ and ${N}^{-}$. A strong TS gene has a big or global impact on expression of genes in cancer. For example, all collected data show that SASH1, STARD13, CBFA2T3, and RECK were strong TS genes (Supplementary Table S2). These TS genes globally influence on expression of genes in cancer, which is supported by gene function annotation and protein structure knowledge. SASH1 and STARD13 contain SAM (sterile alpha motif) domain^31,32^ while the weak TS genes do not have this domain. SAM is an interaction module presented in a wide variety of various proteins and involved in many biological processes. It has homo- and hetero-oligomerise forming multiple self-association architectures and binding to various SAM-domain and non-SAM domain proteins^33^, DNA, and RNA^34^. Proteins with one or more SAM domains broadly regulate RNA transcription and protein translation. SASH1 possessing of two SAM domains helps us to understand why SASH1 was so strong TS gene in all collected data including GSE21933 (Supplementary Fig. S6 and Supplementary Table S2). The effects of strong TS genes SASH1 and STARD13 suggest that if a TS gene has functions in transcription, it could play a global role in expression regulation. This is supported by also another strong TS gene CBFA2T3 (Supplementary Table S2) that encodes a member of the myeloid translocation gene family which interacts with DNA-bound transcription factors and recruits a range of corepressors to facilitate transcriptional repression. These strong TS genes were down-regulated either due to histone methylation (HM) or due to loss of heterozygosity (LOH)^35,36^. Our results show that the strong TS genes SASH1, STARD13, and CBFA2T3 were negatively correlated with up-regulated histone methylation genes SMYD3 and SMYD5 at stage 2 (Supplementary Fig. S9). Furthermore, SASH1 and STARD13 were negatively correlated with all these four up-regulated histone methylation genes detected at stage 3 (Supplementary Fig. S10), and CBFA2T3 was connected with SMYD3, SMYD5 and SUV39H2. Hence, the strong TS genes SASH1, STARD13, RECK, CBFA2T3, RASSF2, RAP1A, and ARHGEF12 can be used as specific biomarkers for diagnosis of NSCLC cancer. Expression correlations of EXT1 with DE genes provide further evidence for this assumption. EXT1 (Exostosin glycosyltransferase 1) encodes a protein that is an endoplasmic reticulum-resident type II transmembrane glycosyltransferase involved in the chain elongation step of heparan sulfate biosynthesis and hence it does not globally regulate expression of the other functional genes. Our results show that it had small $S$-scores and *N*-scores (Supplementary Table S2) in all these collected data and hence EXT1 is a weak TS gene. Interestingly, EXT1 was not connected with histone methylation (HM) genes except for WHSC1 at stage 3. Supplementary Figure S10 suggests that if a TS gene played a stronger role on gene expression, it may be negatively regulated by multiple HM genes. Another example is TS gene PTCH1. In our *S*-score and *N*-score analyses (Figs. 4, 6, Supplementary Figs. S4–S6), PTCH1 was a very weak TS gene in all collected gene expression data (Supplementary Table S2). Biological study shows that PTCH1 is a protein receptor for ligand Sonic Hedgehog. PTCH1 and Sonic Hedgeog matching triggers signals that prevents cells from growing and dividing (proliferating)^37^. So, unlike SASH1, PTCH1 does not impact on expression of multiple other functional genes in mechanics. Likewise, PTCH1 also did not have association with histone methylation genes SMYD3 and SMYD5 at stage 2(Supplementary Fig.S9). These implicate that *S*-scores and *N*-scores can allow one to find another type of biomarkers such as EXT1, PTCH1, KLK10, APC, TRIM13 that have strong information sensitive to early cancer. All results show that stronger TS genes were down-expressed in cancer patients in all collected cohorts and at all tumor stages and had larger ${S}^{+}$ and ${N}^{+}$. This property lets stronger TS genes be used as ideal biomarkers for precision diagnosis and prognosis of cancer patients.

## Methods

### Data collection

Although many datasets related to NSCLC have been published, complete Affymetrix microarray datasets derived from NSCLC adenocarcinoma patients with nonsmoking at stages 1–4 have not yet been available^23^ to date. Recently a microarray experiment with 60 pairs of normal lung tissues and tumor specimens collected from 60 Taiwan nonsmoking female patients at stages 1–3(only one patient at stage 4) was conducted by Lu et al.^1^ on GeneChip Human Genome U133Plus 2.0 expression array using platform GPL570 (Affymetrix, Inc.). The microarray data are available for downloading at http://www.ncbi.nlm.nih.gov/geo/ with access number GES19804^1^. The data consist of 54,675 probes and 59 adenocarcinoma tumor samples, 1 squamous cell carcinoma tumor sample, and 60 normal lung samples at tumor stages 1–4. Specifically, 35 patients were at stage 1, 11 patients at stage 2, 12 patients at stage 3 and one patient at stage 4. To validate that expression of TS genes is suppressed in cancer status, we downloaded three independent datasets GSE18842^27^, GSE40419^28^, and GSE21933^29^ from GEO. GSE18842 is a microarray data derived from Spanish cohort of 45 NSCLC patients. The microarray dataset was created from GeneChip Human Genome using platform GPL570 and composed of 45 normal samples and 45 NSCLC samples. GSE40419 is RNA-seq transcriptomic data from a Korean cohort consisting of 36 adenocarcinoma lung cancer patients without smoking history and 51 adenocarcinoma lung cancer patients in smoking status. GSE21933 is also microarray data generated by using Phalanx Human OneArray chip on platform GPL6254 with 21 NSCLC samples and 21 normal lung samples of Taiwan male patients, of which 7 patients were diagnosed to be at stage 1, 3 at stage 2, 6 at stage 3 and 5 at stage 4 and 11 patients were adenocarcinoma lung cancer and 10 were squamous lung cancer. GSE21933 has 30,968 human genome probes but only 17,819 probes have gene annotation. The RNA transcriptomic sequences analysis was conducted in cancer samples and matching adjacent normal samples ^29^. Since our study used published cohort data available in the public domain, we did not necessarily seek specific ethical reviews and/or consents from the patients of the original studies.

### Quality control of the microarray data

We used two-way scatter plot to visualize data of the two replicate tumor samples and used Pearson correlation coefficient to evaluate the quality of the data.

### Differential expression (DE) analysis

At stage 4 only one patient was recruited and hence the microarray data at stage 4 were removed out from our statistical differential expression analyses. Since in Taiwan female cohort some substages have too few patients, our differential analysis was done at stage level, not at substage level. We here employed RAM^25^ and Bejamini Hochberg multiple test procedure^38^ to compare differences in expressions of genes between the tumor and normal tissue sample at stages 1–3, respectively, and at all these three stages. All differentially expressed genes were identified at FDR < 0.01.

### Classification of tumor stage samples

The principal component analysis (PCA), which reduces higher-dimensional data into three-dimensional components, was used to classify lung cancer stages by using data of 26 TS genes and heatmap was used to visualize differential expression of DE genes between two conditions. The PCA and heatmaps were conducted by using Genesis 1.7.7.

### Heatmaps

In cohort GSE18842, the expression data of the 26 TS genes were retrieved from microarray using probe id. In cohort GSE40419, since the dataset does not have gene probe ids, we used gene names (gene office symbols) to retrieve expression data of 55 RNA isoforms of these 25 TS genes from RPKM dataset. These expression data of TS genes or isoforms were transformed into z-score data. Heatmaps for these z-score data were made by using heatmap.2 in R-environment.

### *S*-scores

If a gene plays an important or central role in tumor development, then the gene would be correlated with many other functional genes in expression. For example, transcriptional regulation factor or post-transcriptional regulation factor regulates expression of a lot of other genes and hence it correlates with these genes in expression. A TS gene that is negatively correlated with a set of genes in expression may have a negative role in expression regulation of these genes while a TS gene that is positively correlated with a set of genes in expression has positive effect on expression of these genes. Based on this principle, we here proposed *S*-scores to measure role of a TS gene in tumor development. Briefly, *S*-scores are represented by ${S}^{+}$,${S}^{-}$ and $S$. ${S}^{+}$ is defined as proportion of sum of correlation coefficients larger than or equal to a given threshold T under Bonferroni adjusted $\alpha$ = 0.05 to total of sums of correlation coefficients > 0:

$${S}_{i}^{+}=\frac{{\sum }_{j=1}^{m}{r}_{ij}|r\ge T}{{\sum }_{j=1}^{m}{r}_{ij}|r>0}$$

where *m* are numbers of DE genes detected in differential expression test. Similarly, ${S}^{-}$ is defined as

$${S}_{i}^{-}=\frac{{\sum }_{j=1}^{m}{r}_{ij}|r\le -T}{{\sum }_{j=1}^{m}{r}_{ij}|r<0}.$$

*S*-score is

$${S}_{i}=\left({\sum }_{j=1}^{m}{r}_{ij}||r|\ge T\right).$$

*S*-score depends on algebra sum of positive and negative correlation coefficients and hence its domain is $(-\infty ,0,+\infty )$. *S* > 0 shows that the TS gene has larger positive effect on down-regulation of genes (positive correlation expression or both a TS gene and DE genes are down-expressed in cancer) than negative effect on up-regulation of genes (negative correlation expression or a TS gene is down-expressed but the DE genes are up-expressed in cancer), inversely, *S* < 0 indicates that the negative effect on up-regulation of gene expression is larger than the positive effect and *S* = 0 implicates that a TS gene has no role in gene expression regulation or balances up-expression and down-expression of the other genes in cancer.

$T=\frac{|t|}{\sqrt{{t}^{2+(n-2)}}}$ is a threshold value where *t* = qt(p,df) where qt is a function converting p to t-value with df where p= $\frac{\alpha }{m}$ (Bonferroni adjusted cutoff probability) and df is degree of freedom (df = (n-2)). In cohort GSE19804, T was calculated to be 0.58 at stage 1, 0.765 at stage 2, and 0.75 at stage 3. In cohort GSE40419, T = 0.538 for nonsmoking status, and 0.45 for smoking status. In cohort GSE18842, T = 0.474.

### *N*-scores

We here define network score or *N*-score as ratio of a number of nodes shared with a TS gene and all other TS genes in a network constructed at a given significance level to that in a network constructed at insignificance level for a TS gene. Let **R** be correlation matrix $n\times{m}$ with element ${r}_{ij}$, i = 1, 2, …, *n* and j = 1, 2 ,…, *m* where *n* is number of TS genes defined and *m* is number of DE genes identified in differential analysis. Let **G** be a gene network matrix $n\times{m}$ with element ${g}_{ij}$ where ${g}_{ij}$=1 if ${r}_{ij}$> T or ${g}_{ij}$= 0 otherwise. Thus, **G** is a bivariable matrix constructed with “0” and “1” where “1” presents a node connecting a TS gene to a DE gene and “0” presents that there is no node between ST and DE genes, that is, a TS gene is not connected to a DE gene at T significance level. In order to know how many nodes a TS gene shares with another TS gene, we need to compare them across all DE genes. Let ${\mathbf{A}}_{{\varvec{i}}}$ be a vector of TS gene *i* from **G** and ${\mathbf{B}}_{{\varvec{k}}}$ be another vector of TS gene *k* from **G** where $i\ne k$. Let ${{\varvec{C}}}_{{\varvec{i}}{\varvec{k}}}^{+}={\mathbf{A}}_{{\varvec{i}}}{\mathbf{B}}_{{\varvec{k}}}$**,** that is, ${c}_{ikj}^{+}={a}_{ij}{b}_{kj}=1$ if ${a}_{ij}=1\ and\ { b}_{kj}=1$ or ${c}_{ikj}^{+}=0$ otherwise where *j* = 1, …, *m*. Therefore, ${{\varvec{C}}}_{{\varvec{i}}{\varvec{k}}}^{+}$ is a node vector shared with TS genes *i* and *k*. Repeat this procession from *k* = 1,…, $k\ne i$,… *k* = n and take maximum ${c}_{ikj}^{+}$ over all *m* DE genes to form a shared node vector for TS gene *i*:$C_i^{+ } = \sum\nolimits_{j = 1}^m {\max_{k \ne i}^m\left( {c_{ikj}^{+}} \right)}$. In a similar way, we get $C_i^{+0} = \sum\nolimits_{j = 1}^m {\max_{k \ne i}^m\left( {c_{ikj}^{+0}} \right)}$ under insignificance level where ${g}_{ij}$=1 if ${r}_{ij}$> 0 or ${g}_{ij}$=0 if ${r}_{ij}\le 0$. The positive *N*-score is defined as ${N}_{i}^{+}=\frac{{C}_{i}^{+}}{{C}_{i}^{+0}}$. Similarly, we also have ${N}_{i}^{-}=\frac{{C}_{i}^{-}}{{C}_{i}^{-0}}$ in which ${g}_{ij}$=1 if ${r}_{ij}$< -T or ${g}_{ij}$= 0 otherwise under significance level of $\alpha$ for defining ${C}_{i}^{-}$ and ${g}_{ij}$=1 if ${r}_{ij}$< 0 or ${g}_{ij}$= 0 if ${{r}_{ij}}{\ge}0$ for defining ${C}_{i}^{-0}.$

### Gene sets enrichment analysis (GSEA)

To explore if the 26 TS genes used as a gene set co-work or co-act in biological functions or pathways, we performed GSEA analysis^30^ of the 26 TS genes using microarray from Taiwan female cohort (GES19804) as gene expression dataset associated with the fold changes corresponding to their differential expression between normal and tumors samples. In GSEA analysis, we setup these 26 TS genes selected from GO analysis of differentially expressed genes as a gene set data, used normal and tumor samples to make phenotype dataset. We selected Human_AFFY_HG_U133_ MSigDB.v7.1.chip as annotation. GSEA analysis was respectively performed on microarray datasets at stages 1, 2, 3 and all stages. Phenotype and gene permutations were respectively performed each for 1000 times for calculating p-value.

## Supplementary Information

Supplementary Legends.

Supplementary figure S1.

Supplementary figure S2a.

Supplementary figure S2b.

Supplementary figure S2c.

Supplementary figure S3.

Supplementary figure S4.

Supplementary figure S5a.

Supplementary figure S5b.

Supplementary figure S6a.

Supplementary figure S6b.

Supplementary figure S7.

Supplementary figure S8.

Supplementary figure S9.

Supplementary figure S10.

Supplementary table S1.

Supplementary table S2.
