# Supplementary material for: Use tumor suppressor genes as biomarkers for diagnosis of non-small cell lung cancer
Source: Sci Rep. 2021 Feb 12;11:3596. doi: 10.1038/s41598-020-80735-x (PMC7881207; doi:10.1038/s41598-020-80735-x)
Supplement: Supplementary file 1 — Supplementary Legends. [file 41598_2020_80735_MOESM1_ESM.docx]

Use tumor suppressor genes as biomarkers for diagnosis of non-small cell lung cancer

Chuantao Zhang^1#,^ Man Jiang^1#^, Na Zhou^1^, Helei Hou^1^, Tianjun Li^1^, Hongsheng Yu^1^, Yuan-De Tan^2*^, [Xiaochun](mailto:zhangxiaochun9670@126.com%EF%BC%8CXiaochun) Zhang^1*^

1: Precision Medicine Center of Oncology, the Affiliated Hospital of Qingdao University, Qingdao, 266003, China. 2: Dan L Duncan Comprehensive Cancer Center, Baylor College of Medicine, Houston, Texas, 770303

#equal first author and * corresponding authors

**Figure S1. Microarray data quality**

Two-replicate scatter plot is used to visually display quality of microarray data. If one sample is replicate of another sample, then they are sampled from a distribution and two-replicate scatter plot would display the dots falling in area around a positive diagonal line with high Pearson correlation coefficient (r ≥ 0.9), otherwise, the dots falling into the area way from diagonal line. A high-quality dataset would have good repeatability. A: samples on x-axis and y axis are two adjacent replicate samples1 and 2 drawn from stage IA. B: samples on x-axis and y-axis are another adjacent replicate samples7 and 8 drawn from stage IA. C: samples on x-axis and y-axis are two adjacent replicate samples 3 and 4 drawn from stage IIA. D: samples on x-axis and y-axis are two adjacent replicate samples 3 and 4 drawn from stage IIB. E: samples on x-axis and y-axis are two adjacent replicate samples 1 and 2 drawn from stage IIIA. F: samples on x-axis and y-axis are two adjacent replicate samples 1 and 2 drawn from stage IIIB.

**Figure S2. Differential expression of tumor suppressor genes**

Differential expression of 20 TS genes GPC3, KLK10, KCNRG, RHOB, STARD13, CDKN1C (a), EXT1, LIMD1, DAB2IP, DDX5, SASH1, MCC (b), LATS2, RAP1A, FOXP1, TBRG1, PIK3CA, DCC (c) between normal and cancer samples at stages 1, 2 and 3 in Taiwan female breast cancer cohort (**GSE19804**) was displayed by boxplots where p-value is given for t-test for expression difference of TS gene between normal and cancer samples.

**Figure S3. Heatmaps of TS genes in cohorts GSE18842 and GSE40419**

**GSE18842**^25^ is a Spain cohort consisting of 45 non-small cell lung cancer (NSCLC) samples and 45 normal tissue samples. Microarray for gene expression analysis was performed in **GSE18842**^25^. Since there are no stages in this cohort, heatmap of 25 TS genes for differential expression was made between 45 normal samples and 45 cancer samples. **GSE40419^26^** is count data of RNA-seq reads mapping to human hg19. **GSE40419^26^** has two sub-datasets: one consists of 36 cancer samples and 27 normal samples from patients without smoking history and the other has 50 cancer samples and 50 normal samples from 50 smoking patients. In this RNA-seq data, most of these 25 TS genes have more than one isoform. Blue color denotes lower expression and red color presents higher expression and white color show no difference between normal and cancer samples. Heatmap values of genes are z-cores.

**Figure S4. Histogram plots for** $\boldsymbol{S}^{\boldsymbol{(+)}}\boldsymbol{,}\boldsymbol{S}^{\boldsymbol{(-)}}$ **and S of TS genes in cohorts GSE18842 and GSE40419**

TS genes with negative correlation coefficients $\leq$ -T (T is a threshold value with Bonferroni adjusted p < 0.05, see Materials and Methods section for calculation of T value) have a negative role that up-regulates expression of genes while those with positive correlation coefficients$\geq$ T have a positive role that suppresses expression of the other genes. S-score is defined as sum of all correlation coefficients of a TS gene with the other differentially expressed (DE) genes larger or less than a given threshold: $S_{i}$ = $\sum_{j=1}^{m} r_{ij}||r|\geq T$ where *m* is number of DE genes and n is number of TS genes. $S^{(+)}$ is proportion of sum of all correlation coefficients of a TS gene with all DE genes larger than or equal to a given threshold to sums of correlation coefficients larger than zero: $S_{i}^{(+)}=(\sum_{j=1}^{m} r_{ij}|r\geq T)/(\sum_{j=1}^{m} r_{ij}|r>0)$ . Similarly, $S^{(-)}$ is defined as $S_{i}^{(-)}=(\sum_{j=1}^{m} r_{ij}|r<-T)/(\sum_{j=1}^{m} r_{ij}|r<0)$ . T = 0.474 for GSE18842^25^, 0.538 for nonsmoking **GSE40419^26^** cohort, and 0.45 for smoking **GSE40419^26^** cohort.

**Figure S5. Histogram plots for** $\boldsymbol{N}^{\boldsymbol{(+)}}$**and** $\boldsymbol{N}^{\boldsymbol{(-)}}$ **of TS genes in GSE18842 and GSE40419**

N-score is defined as ratio of node number shared with a TS gene and all the other TS genes in a network constructed at a given significant level (for example, T) to that in a network constructed at insignificance level(r > 0 or r < 0). N-score is given by $N^{(+)}$ and $N^{(-)}$(see Materials and Methods for detail definition and calculation). $N^{(+)}$ is used to measure accordant effects of a TS gene in a positive correlation networks on down-expression of genes. $N^{(-)}$is used to measure discordant effects of a TS gene in a negative correlation networks on up-expression of genes. Here significant correlation network was constructed under T = 0.474 in **GSE18842**^25^ and built under T = 0.538 in nonsmoking **GSE40419^26^** cohort. 25 TS genes were ranked by sorting $N^{(+)}$ and $N^{(-)}$ values from the smallest to the largest. In these two cohorts, TS genes with $N^{(+)}$> 0.4 and/or $N^{(-)}$ > 0.2 are called strong TS genes.

**Figure S6. Histogram plots for**$\boldsymbol{S}^{\boldsymbol{(+)}}\boldsymbol{,}\boldsymbol{S}^{\boldsymbol{(-)}}$**, S,** $\boldsymbol{N}^{\boldsymbol{(+)}}$**and** $\boldsymbol{N}^{\boldsymbol{(-)}}$ **of TS genes in GSE21933**

To verify generality of results obtained from $S^{(+)},S^{(-)}$, S, $N^{(+)}$and $N^{(-)}$ analyses of TS genes in **GSE19804**^1^, **GSE18842**^25^, and **GSE40419^26^**, we retrieved sub-data of tumor samples at stages 2 and 3 (stages are known in such data) and 21 normal sample from **GSE21933^27^** and performed two-sample t-test between tumor and normal samples and then used Bejamin-Hochberg procedure^33^ to adjust p-value. We retrieved data of these 26 TS genes from sub-dataset of stages 2 and 3 and normal samples and data of DE genes identified, and used these two datasets to construct correlation network under T=0.538. We calculated $S^{(+)},S^{(-)}$, S, $N^{(+)}$and $N^{(-)}$ and ranked the TS genes by sorting these scores and made histogram plots of $S^{(+)},S^{(-)}$, S in Figure S6a and of $N^{(+)}$and $N^{(-)}$ in Figure S6b.

**Figure S7. Comparison of** $\boldsymbol{S}^{\boldsymbol{(+)}}$**and** $\boldsymbol{S}^{\boldsymbol{(-)}}$ **and** $\boldsymbol{N}^{\boldsymbol{(+)}}$**and** $\boldsymbol{N}^{\boldsymbol{(-)}}$ **accumulation profiles among GSE40419, GSE18842 and GSE21933**

Accumulations of N-scores and S-scores are calculated by $X_{1}^{(a)}=N_{1}^{(a)}$, $X_{2}^{(a)}=N_{1}^{(a)}+N_{2}^{(a)}$, …, $X_{n}^{a}=N_{1}^{(a)}+N_{2}^{(a)}+\cdots+N_{n}^{(a)}$and $Y_{1}^{(a)}=S_{1}^{(a)}$, $Y_{2}^{(a)}=S_{1}^{(a)}+S_{2}^{(a)}$, …, $Y_{n}^{(a)}=S_{1}^{(a)}+S_{2}^{(a)}+\cdots+S_{n}^{(a)}$ where $a$ = “+” or “-”.$N_{1}^{(a)}\leq N_{2}^{(a)}\leq\cdots\leq N_{n}^{(a)}$ and $S_{1}^{(a)}\leq S_{2}^{(a)}\leq\cdots\leq S_{n}^{(a)}$ are respectively ranked *N*-scores and *S*-scores from the smallest to the largest. Here, 1, 2, …, n in X or Y are numbers of TS genes. The profiles of S-score and N-score accumulation in **GSE40419^26^** are similar to those at stage3 in **GSE19804**^1^. So, cancer patients in **GSE40419^26^** (Figure 7) is inferred to be at stage3. The profiles of S-score and N-score accumulation in **GSE21933^27^** were obtained from stage 2 and 3. Difference between accumulations $X^{(+)} \mathrm{and} X^{(-)}$ or accumulations $Y^{(+)} \mathrm{and} Y^{(-)}$ in **GSE18842**^25^ is smaller than that in **GSE21933^27^** but larger than that at stage 2 in **GSE19804**, suggesting that most of the cancer patients in **GSE18842**^25^ were at stage2 but small part of patients was at stage 3.

**Figure S8. GSEA analysis of TS genes**

GSEA^28^ plot shows profile of the running ES score & positions of GeneSet members on the rank ordered List. 26 TS genes selected from GO analysis of differentially expressed genes were setup as a gene set, samples from Taiwan female breast cancer cohort were used as phenotype data and Human_AFFY_HG_U133_ MSigDB.v7.1.chip was used as annotation file. GSEA analysis was respectively performed on microarray datasets at stages 1, 2, 3 and all stages. Permutation for calculation of p-values was performed on gene set for 1000 times. Stage 1: 38 normal samples and 15 cancer samples. Stage 2: 38 normal samples and 11 cancer samples. Stage 3: 38 normal samples and 7 cancer samples. All stages: 38 normal samples and 38 cancer samples.

**Figure S9. Network of TS genes with two histone methylation genes detected at stage 2**

The network of two histone methylation genes SMYD3 and SMYD5 detected by differential expression test with 23 TS genes detected at stage 2 was constructed with correlation coefficients < - 0.6.

**Figure S10. Network of TS genes and histone methylation genes detected at stage3**

The circle degree network was used to visualize relationship between histone methylation genes SMYD3, SMYD5, SUV39H2, and WHSC1 detected at stage 3 and 26 TS genes. Degree of node sizes and colors denotes numbers of connects between histone methylation genes and TS genes. The network was constructed with correlation coefficients < - 0.6.
