# Supplementary figures and images for: Use tumor suppressor genes as biomarkers for diagnosis of non-small cell lung cancer
Source: Sci Rep. 2021 Feb 12;11:3596. doi: 10.1038/s41598-020-80735-x (PMC7881207; doi:10.1038/s41598-020-80735-x)

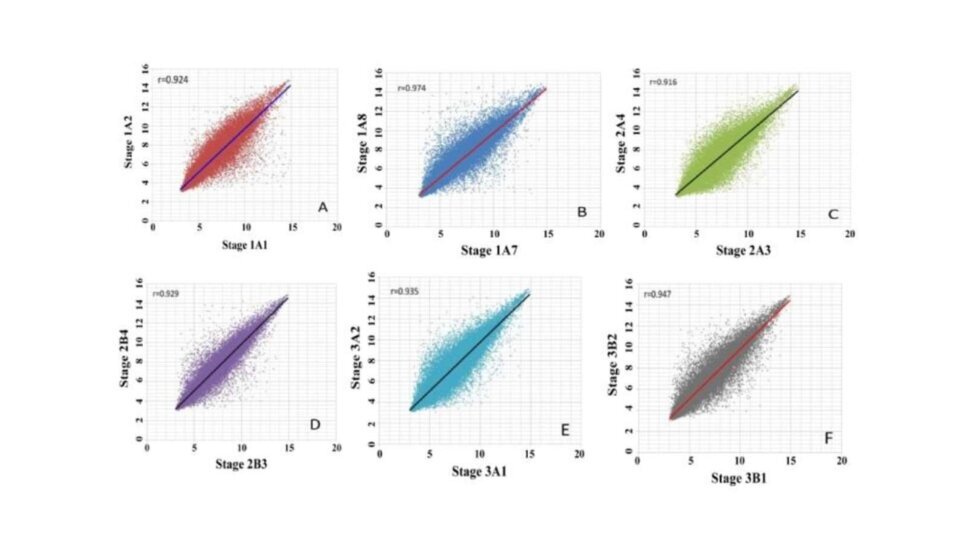

Supplement: Supplementary file 2 — Supplementary figure S1. [file 41598_2020_80735_MOESM2_ESM.jpg]

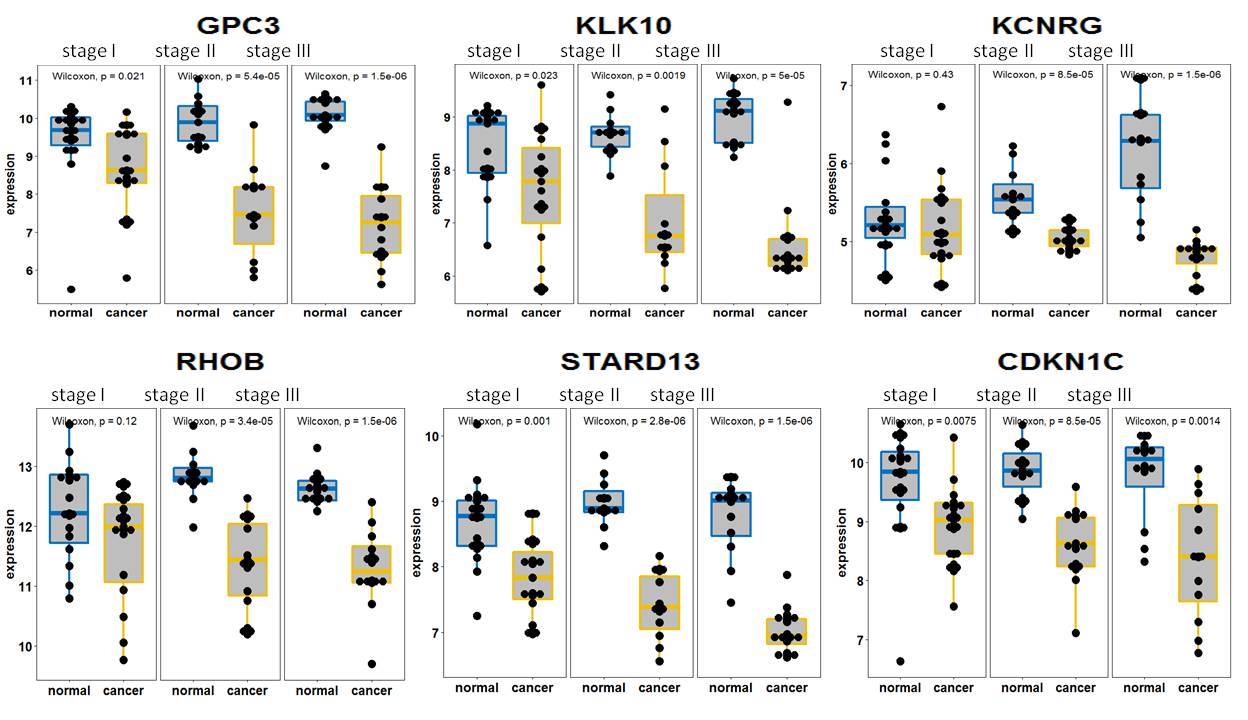

Supplement: Supplementary file 3 — Supplementary figure S2a. [file 41598_2020_80735_MOESM3_ESM.jpg]

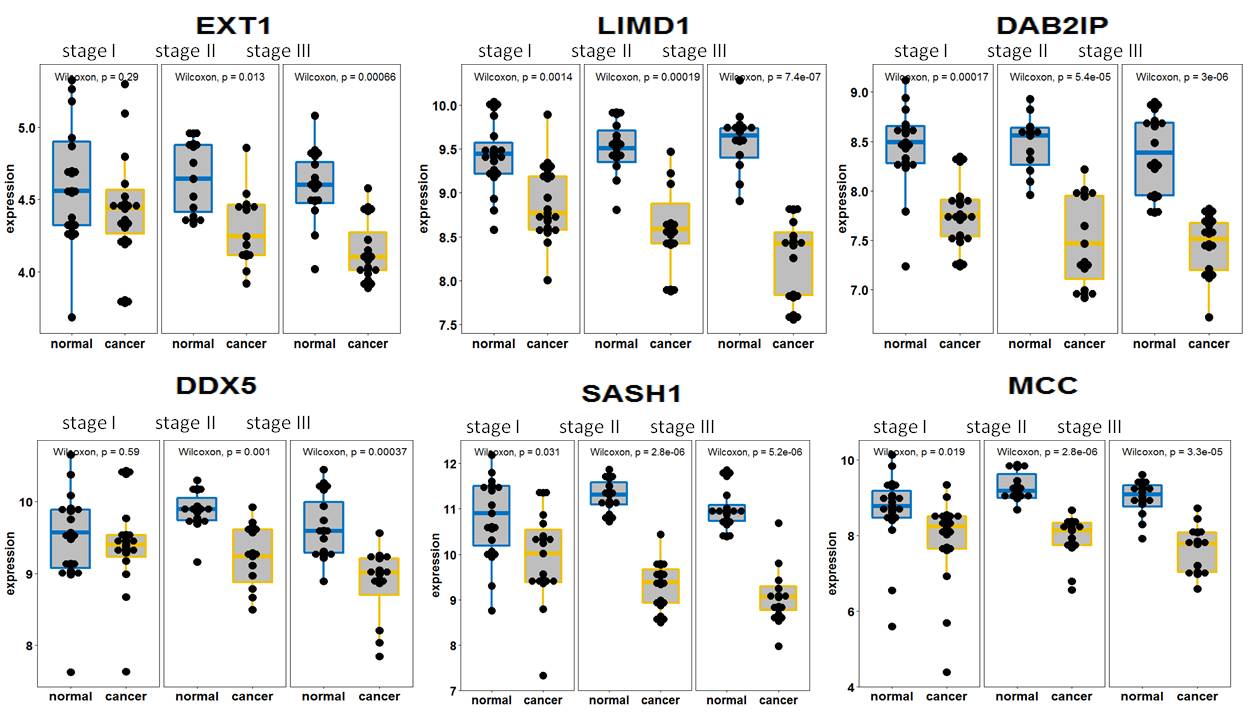

Supplement: Supplementary file 4 — Supplementary figure S2b. [file 41598_2020_80735_MOESM4_ESM.jpg]

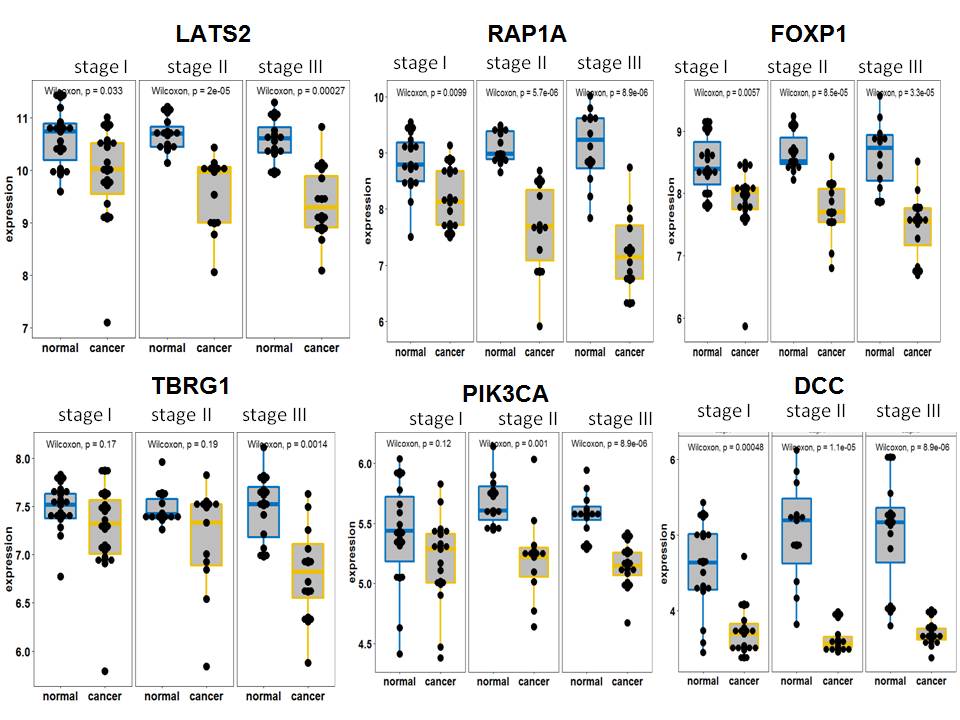

Supplement: Supplementary file 5 — Supplementary figure S2c. [file 41598_2020_80735_MOESM5_ESM.jpg]

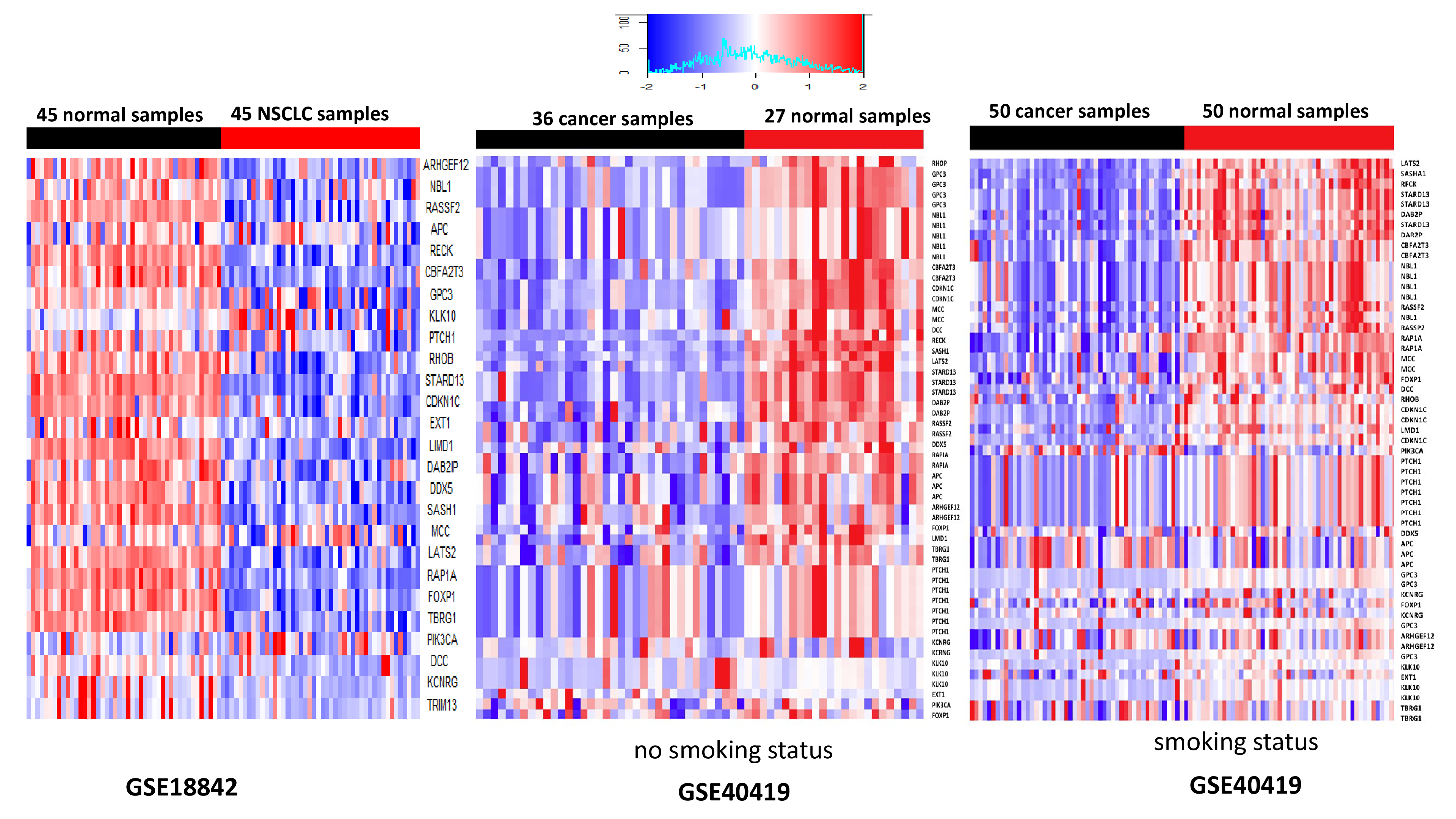

Supplement: Supplementary file 6 — Supplementary figure S3. [file 41598_2020_80735_MOESM6_ESM.jpg]

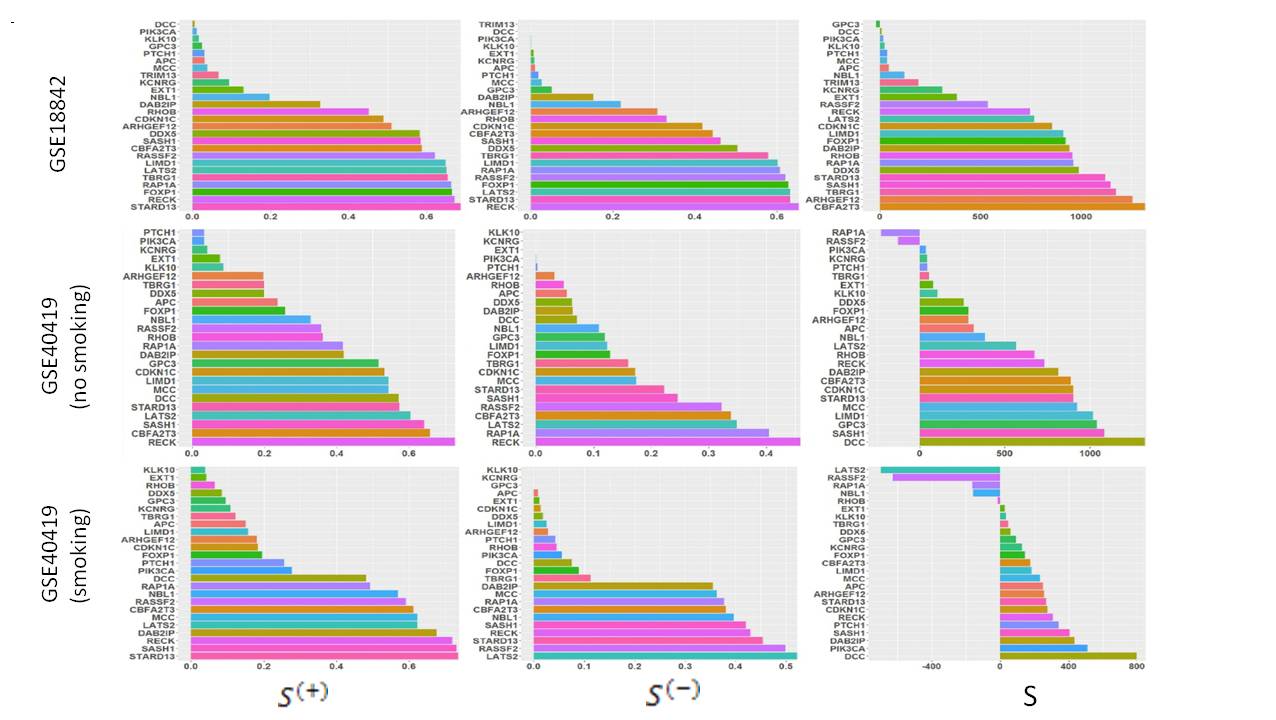

Supplement: Supplementary file 7 — Supplementary figure S4. [file 41598_2020_80735_MOESM7_ESM.jpg]

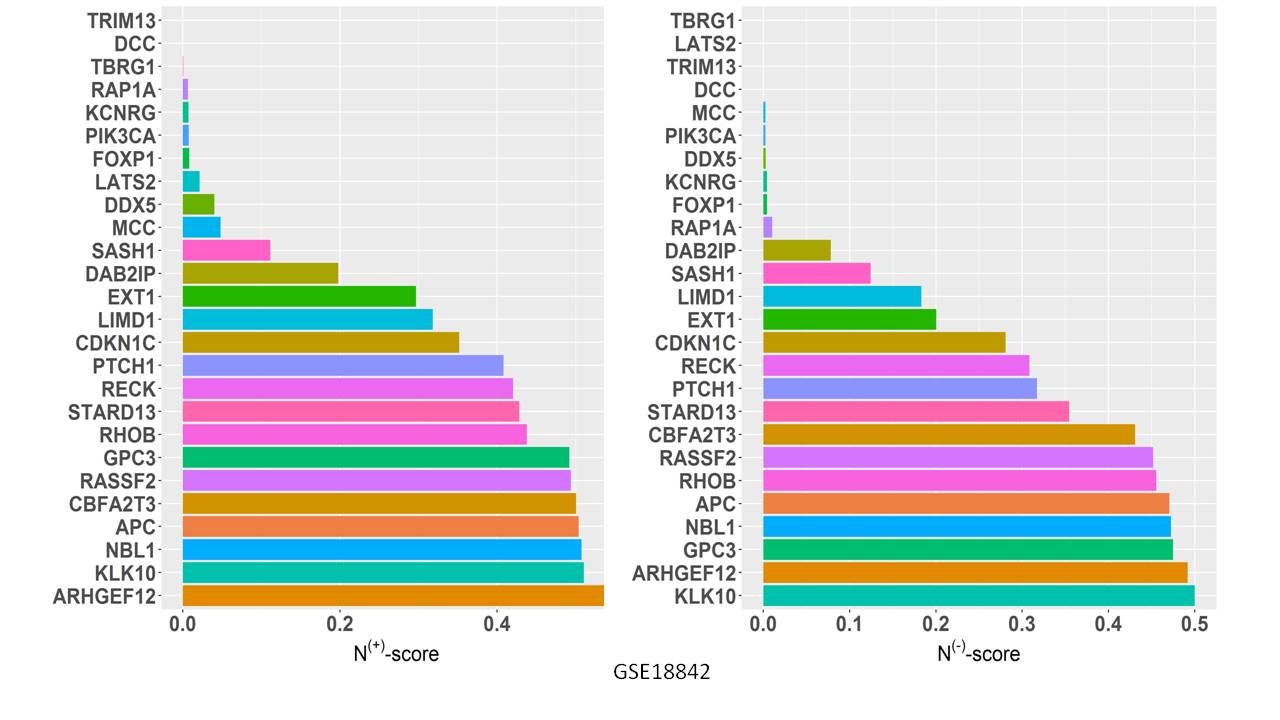

Supplement: Supplementary file 8 — Supplementary figure S5a. [file 41598_2020_80735_MOESM8_ESM.jpg]

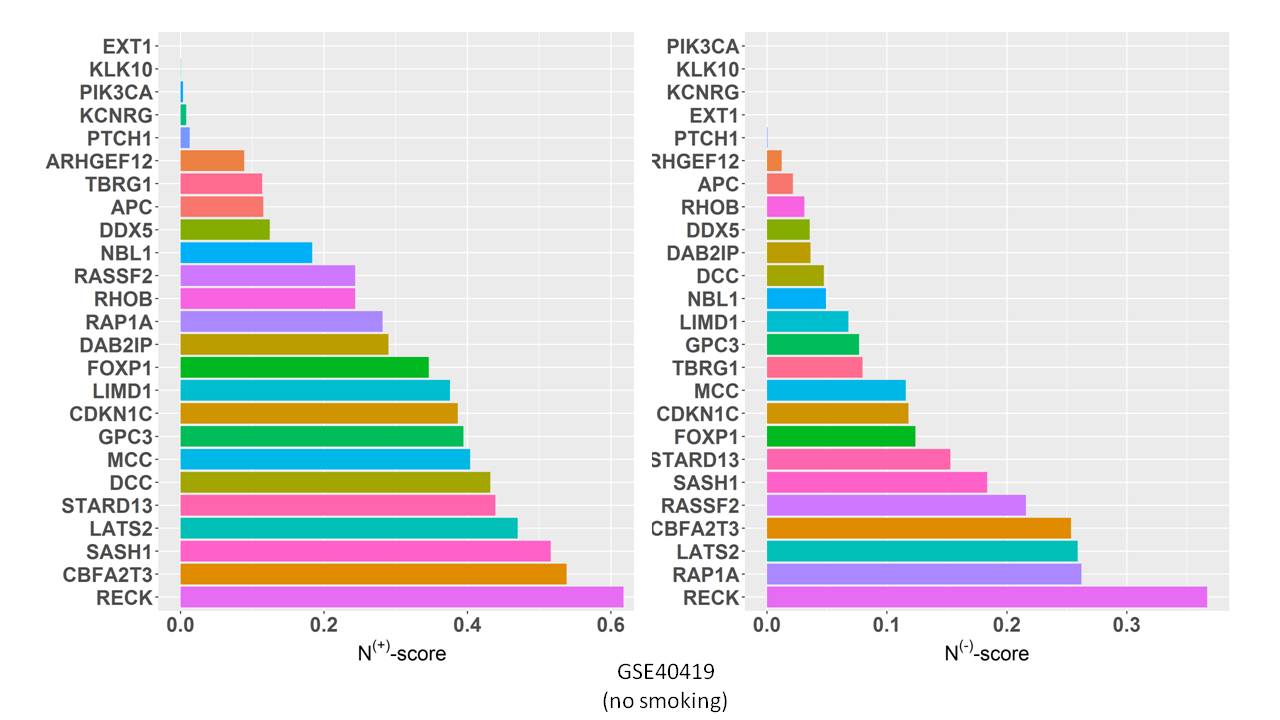

Supplement: Supplementary file 9 — Supplementary figure S5b. [file 41598_2020_80735_MOESM9_ESM.jpg]

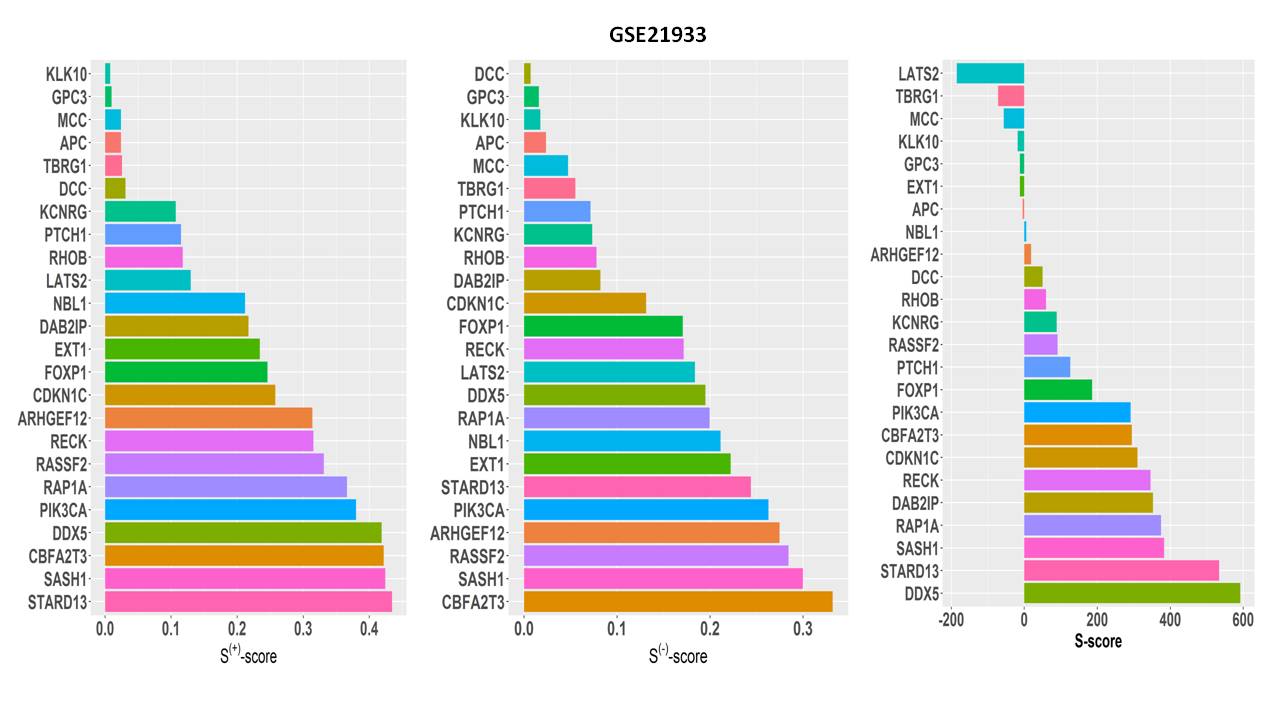

Supplement: Supplementary file 10 — Supplementary figure S6a. [file 41598_2020_80735_MOESM10_ESM.jpg]

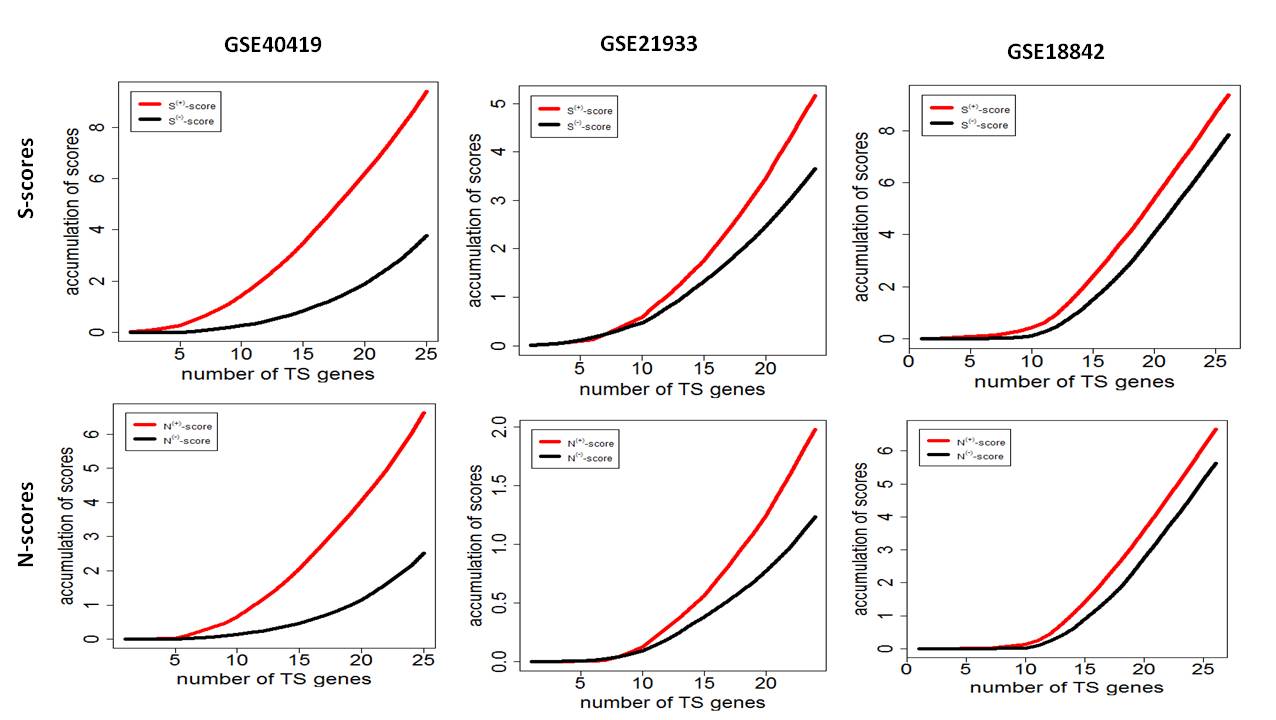

Supplement: Supplementary file 11 — Supplementary figure S6b. [file 41598_2020_80735_MOESM11_ESM.jpg]

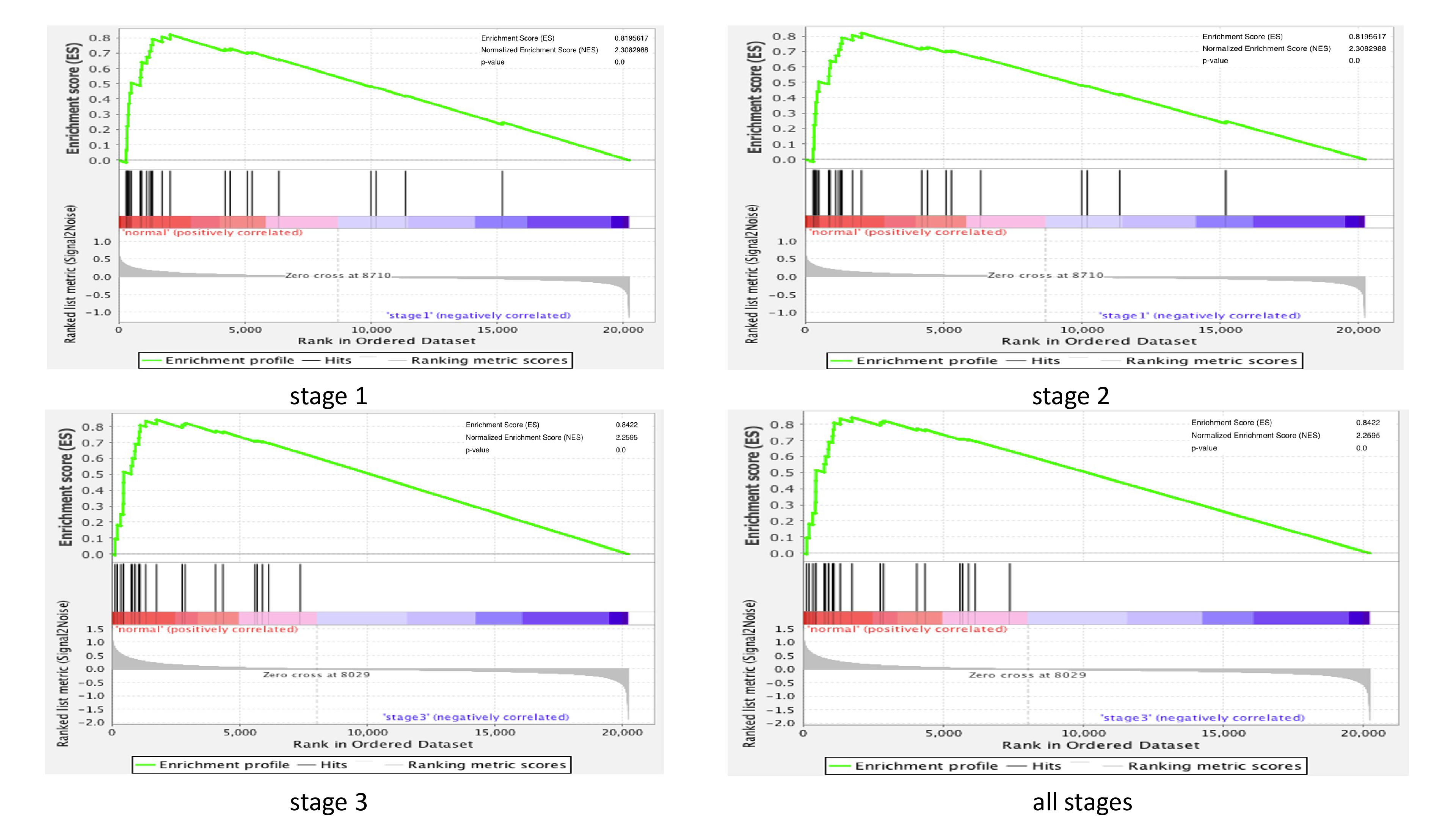

Supplement: Supplementary file 12 — Supplementary figure S7. [file 41598_2020_80735_MOESM12_ESM.jpg]

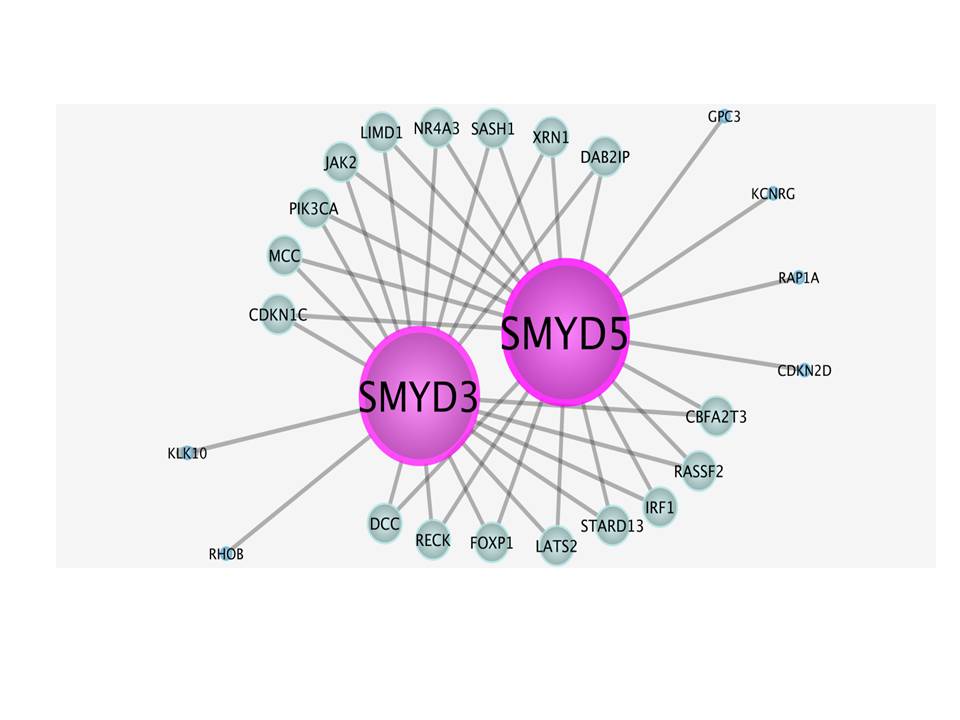

Supplement: Supplementary file 13 — Supplementary figure S8. [file 41598_2020_80735_MOESM13_ESM.jpg]

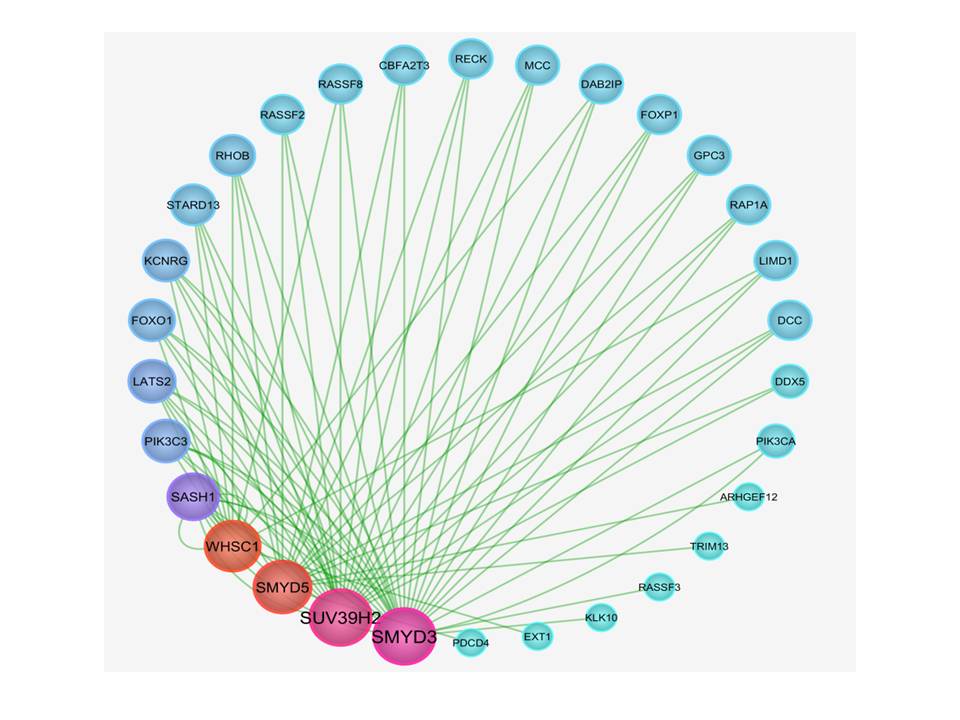

Supplement: Supplementary file 14 — Supplementary figure S9. [file 41598_2020_80735_MOESM14_ESM.jpg]
